# Metastable Oxygen-Induced Light-Enhanced Doping in Mixed Sn–Pb Halide Perovskites

**DOI:** 10.1021/jacs.4c08924

**Published:** 2024-11-04

**Authors:** Jasmeen Nespoli, Matthijs Mugge, Lara M. van der Poll, Snigdha Lal, Bahiya Ibrahim, Bart Boshuizen, Valentina M. Caselli, Arjan J. Houtepen, Lars J. Bannenberg, Tom J. Savenije

**Affiliations:** † Department of Chemical Engineering, Faculty of Applied Sciences, Delft University of Technology, 2629 HZ Delft, The Netherlands; ‡ Department of Radiation Science and Technology, Faculty of Applied Sciences, Delft University of Technology, 2629 JB Delft, The Netherlands

## Abstract

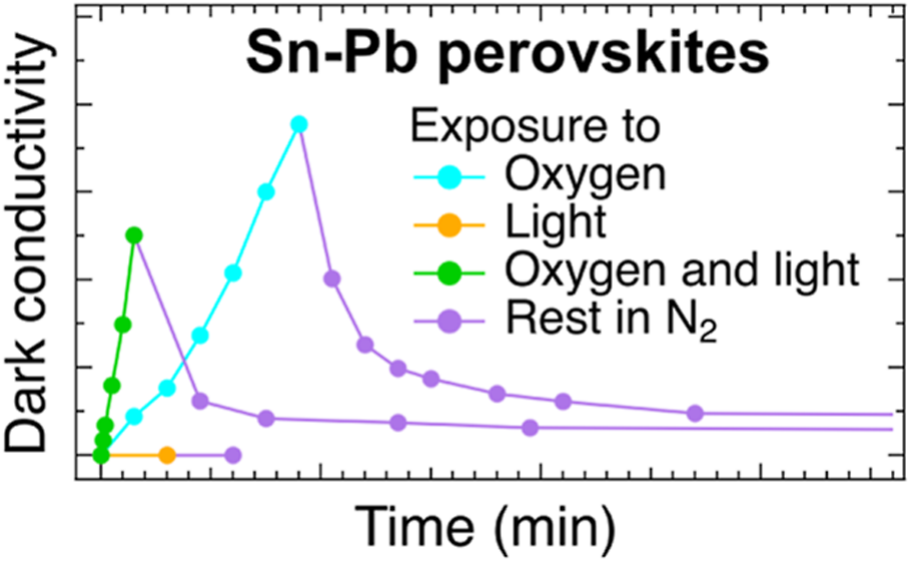

Mixed Sn–Pb halide perovskites are promising absorber materials for solar cells due to the possibility of tuning the bandgap energy down to 1.2–1.3 eV. However, tin-containing perovskites are susceptible to oxidation affecting the optoelectronic properties. In this work, we investigated qualitatively and quantitatively metastable oxygen-induced doping in isolated ASn_*x*_Pb_1–*x*_I_3_ (where A is methylammonium or a mixture of formamidinium and cesium) perovskite thin films by means of microwave conductivity, structural and optical characterization techniques. We observe that longer oxygen exposure times lead to progressively higher dark conductivities, which slowly decay back to their original levels over days. Here oxygen acts as an electron acceptor, leading to tin oxidation from Sn^2+^ to Sn^4+^ and creation of free holes. The metastable oxygen-induced doping is enhanced by exposing the perovskite simultaneously to oxygen and light. Next, we show that doping not only leads to the reduction in the photoconductivity signal but also induces long-term effects even after loss of doping, which is thought to derive from consecutive oxidation reactions leading to the formation of defect states. On prolonged exposure to oxygen and light, optical and structural changes can be observed and related to the formation of SnO_*x*_ and loss of iodide near the surface. Our work highlights that even a short-term exposure to oxygen immediately impairs the charge carrier dynamics of the perovskite, while structural perovskite degradation is only noticeable upon long-term exposure and accumulation of oxidation products. Hence, for efficient solar cells, exposure of mixed Sn–Pb perovskites to oxygen during production and operation should be rigorously blocked.

## Introduction

Over the past years, metal halide perovskites (MHPs), denoted with chemical formula ABX_3_, have been placed in the spotlight for their manifold possible applications,^1−4^ including photovoltaics.^1,5^ Mixed Sn–Pb perovskites, with alternating Sn^2+^ and Pb^2+^ at the B-sites, have emerged as low bandgap absorbers with *E*_g_ = 1.2–1.6 eV in perovskite solar cells.^6,7^ The A-sites can be occupied by methylammonium (MA^+^), CH_3_NH_3_^+^, formamidinium (FA^+^), HC(NH_2_)_2_^+^, cesium, Cs^+^, or a mixture of them, while iodide, I^–^, is commonly chosen for the X-sites. Application of these mixed MHPs in multijunction solar cells is expected to surpass the Shockley–Queisser power conversion efficiency (PCE) limit.^7^ At present, the record PCE is 23.6% for single-junction solar cells and 28.0% for all-perovskite tandem devices containing mixed Sn–Pb perovskites.^8,9^ Furthermore, a partial substitution with tin would reduce the toxicity of fully Pb-based perovskite solar cells, given that lead is potentially hazardous for humans and the environment.^10^

Despite these positive aspects, studies have shown that both intrinsic and extrinsic factors can result in self-doping of tin-containing perovskites.^7,11−13^ This is often related to the oxidation of Sn^2+^ to Sn^4+^.^14,15^ As reported in the literature for pure Sn-based perovskites, during synthesis tin oxidation favors the formation of tin vacancies (V_Sn_), which in combination with iodide interstitials (I_i_) leads to electron acceptor defect levels just below the valence band maximum. As a consequence, it is claimed that valence band electrons occupy these defect states, leading to p-type doping.^7,11,16^ p-Type doping also takes place in the presence of oxygen, which acts as an electron acceptor leading to tin oxidation from Sn^2+^ to Sn^4+^.^13,17−21^

High doping densities are expected to reduce the photogenerated charge carrier mobilities and lifetimes by, respectively, enhanced scattering with ionized impurities and pseudomonomolecular recombination with the background holes. Furthermore, the presence of background free holes not only negatively affects the photovoltaic performance, but may also lead to perovskite degradation and limits the stability of solar cells.^7,11,18,19,22−24^ Studies have focused on the effects of dry or ambient air, humidity and temperature on pure Sn-based perovskites and on mixed Sn–Pb perovskites, both at the material and device level.^18,25−30^ Generally, exposure to ambient air is associated in the literature with an increase in p-type doping due the reaction between tin and oxygen.^13,18^ In such reaction, Sn^2+^ is oxidized to Sn^4+^ by oxygen and free holes are generated leading to p-type doping,^13,31,32^ as shown by eq 1, expressed in Kröger-Vink notation.
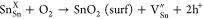
1Equation 1 can be interpreted as the transfer of two electrons from Sn_Sn_^X^ to O_2_, leading to the formation of Sn_Sn_^••^ and O_i_^″^ ions.^20,21^ As a result, Sn_Sn_^X^ ions are removed from the crystal lattice creating tin vacancies, V_Sn_^″^, while SnO_2_ is formed at the surface and two holes are released in the bulk, leading to p-doping.^14^ Tin oxidation most likely occurs at the surface of the perovskite film or grain boundary, where Sn_Sn_^••^ is stable and creates deep electron traps.^11^ On the other hand, tin oxidation may also occur in the bulk, where Sn_Sn_^••^ is claimed to be unstable. Thus, in that case, it is most likely excluded from the perovskite crystal lattice and displaced at the surface.^11,15^

The photostability of Sn-containing perovskites has barely been addressed,^26,28,29^ however mostly for devices.^19,30^ Moreover, only a few reports discuss the effect of oxygen or the combination of oxygen and light specifically on the perovskite optoelectronic properties.^18,28,29^ Although the degradation mechanisms are not fully elucidated, a range of reaction products has been reported, including AX, SnO_2_, SnX_2_, SnX_4,_ PbX_2_, X_2_ and A_2_SnX_6_ (where A = MA^+^, FA^+^, Cs^+^ and X = I^–^).^18,25−30^ Moreover, it is unclear whether the same degradation mechanisms known to exist in pure Sn-based perovskites also apply to mixed Sn–Pb perovskites. Finally, several additives, such as SnF_2_,^13,22,24,33−37^ metallic Sn powder,^38,39^ reducing agents and others,^6,30,40−42^ have been used to suppress the spontaneous or externally triggered doping, even though the precise role of these additives in improving the perovskite properties needs to be fully clarified.

**Scheme 1:**
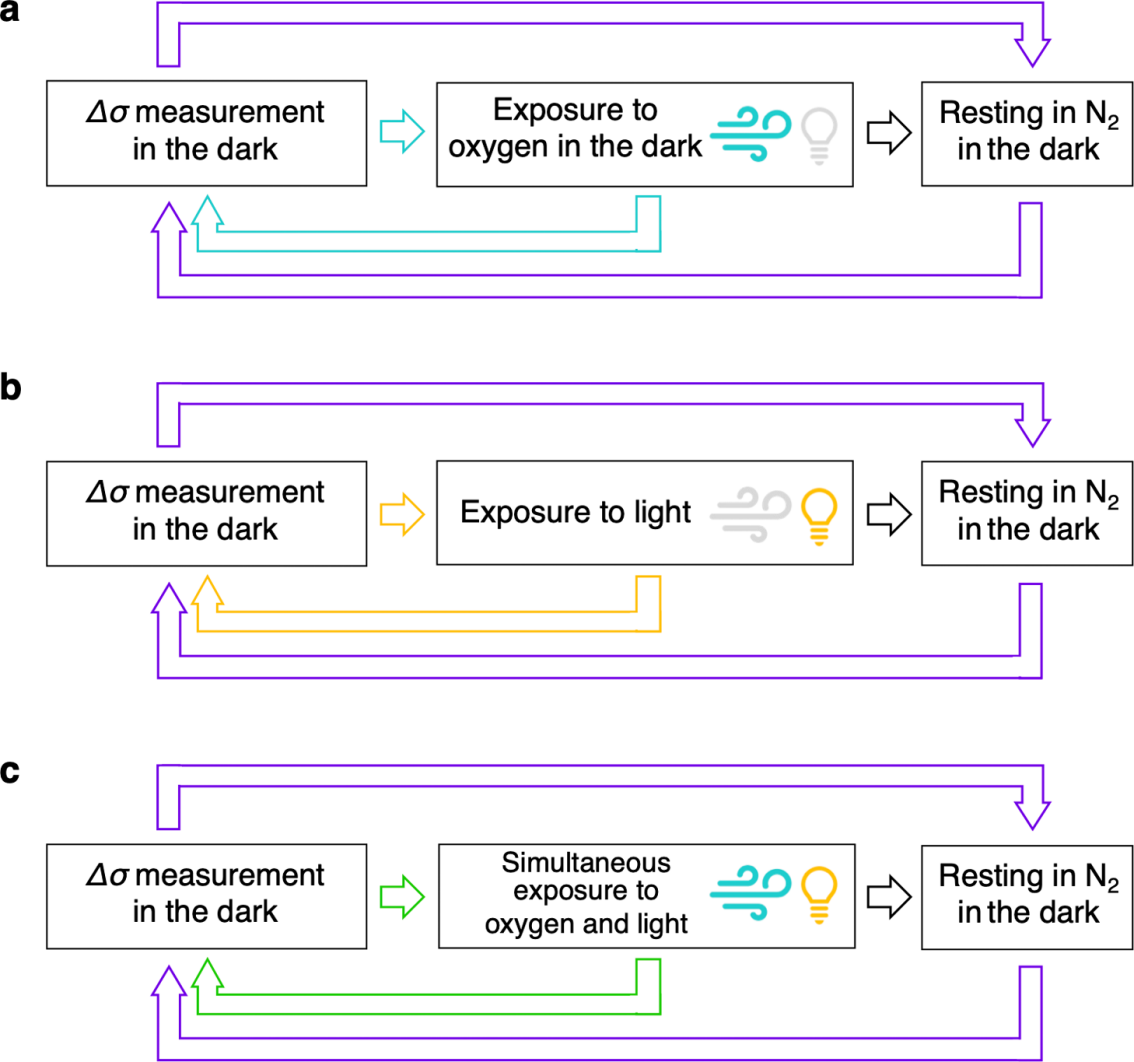
SSMC Measurement Cycles of Perovskite Thin Films Exposed to (a) Only Oxygen (Light Blue Cycle), (b) Only Light (Yellow Cycle) and (c) Simultaneously Oxygen and Light (Green Cycle) for Varying Time Intervals. All SSMC Measurements Were Carried out in the Dark to Discard the Contribution of the Photoconductivity due to the Lamp Light. After a Series of Exposures to Oxygen or/and Light, the Films Were Stored in N_2_ in the Dark and Δσ_dark_ was Measured after Varying Time Intervals (Purple Cycles)

In this work, we investigated the respective effects of exposure to oxygen, light and the combination of both on Cs_0.25_FA_0.75_Sn_*x*_Pb_1–*x*_I_3_ thin films, with 20 mol % SnF_2_ added to the tin precursor solution. First, we inspected the dark conductivity under N_2_ for the pristine perovskite films by means of electrodeless steady-state microwave conductance (SSMC). Next, we studied in a qualitative and quantitative way the effects of the exposure to oxygen, to light and finally to the combination of both on the background conductivity, Δσ_dark_, following the oxidative process as it occurs in the isolated perovskite layer over time. All the different types of exposure cycles and the respective SSMC measurement procedures on each sample are depicted in Scheme 1. Next, we examined the photogenerated charge carrier dynamics by electrodeless laser-pulse induced time-resolved microwave conductivity (TRMC) and the quasi-Fermi level splitting (QFLS) by means of the microwave conductivity setup of the isolated perovskite films (i) under pristine conditions, (ii) directly after exposure and (iii) after storage under N_2_. In this way, without fabricating full perovskite solar cells, which would lead to uncertainty in pinpointing the oxidation mechanism due to the presence of many layers and interfaces, we are able to link the changes in the perovskite absorber to the performance of a corresponding device. To explain the oxygen-induced and light-enhanced doping in Cs_0.25_FA_0.75_Sn_0.5_Pb_0.5_I_3_, structural and optical characterization is performed after prolonged exposure. Our results reveal key insight into the degradation processes in mixed Sn–Pb perovskites, which we use to develop suggestions to tackle these problems and improve the performance and lifespan of mixed Sn–Pb perovskite solar cells.

## Results and Discussion

Mixed Sn–Pb perovskites thin films of composition Cs_0.25_FA_0.75_Sn_*x*_Pb_1–*x*_I_3_ with different tin fractions of *x* = 0.0, *x* = 0.2, *x* = 0.5 and *x* = 1.0, indicated hereafter as Sn_*x*_Pb_1–*x*_, were prepared by spin-coating using anisole as antisolvent, as detailed in the Supporting Information, E/M S1 and S2. The crystal phase and the optical absorption properties of the polycrystalline perovskite films with varying Sn/Pb ratio under pristine conditions were studied by X-ray diffraction (XRD) and ultraviolet–visible–near-infrared spectroscopy (UV–vis–NIR). The results are provided in the Supporting Information. The XRD diffraction patterns in Figure S16 present the characteristic peaks of the perovskite pseudocubic crystal phase. The absorption spectra, shown in Figure S20, reveal bandgap energies following a bowing behavior with varying Sn/Pb ratio. The Sn_0.5_Pb_0.5_ perovskite has the smallest bandgap of the series, amounting to a *E*_g_ ∼ 1.24 eV, in line with the reported values for similar perovskite compositions.^16,24,43−45^

First, we studied the background conductivity, σ_dark_, of the pristine thin films Sn_0.5_Pb_0.5_, measured in the dark in N_2_ by means of SSMC. This method, relying on the interaction of microwaves with free mobile charge carriers, enables the determination of the dark conductivity of thin films in an electrodeless fashion. The working principle and instrumental setup are described in the Supporting Information E/M S4. The frequency scans of all pristine mixed Sn–Pb perovskite layers are compared to a scan of a bare, identical quartz substrate (see Figure S1). In case the perovskite is doped the corresponding σ_dark_ would lead to enhanced microwave absorption, which results in a deepening of the resonance frequency dip. Since all perovskite films showed frequency scans nearly identical to that of quartz, we can conclude that the pristine layers present a relatively low initial background conductivity, σ_0,dark_. As detailed in the Supporting Information E/M S4, calculations imply that for all pristine films σ_0,dark_ ≲ 1 S m^–1^. We attribute the low σ_0,dark_ to the use of SnF_2_ as additive to the spin-coating precursor solution, which is claimed to reduce tin oxidation and hinder the formation of tin vacancies during the synthesis process, associated to doping of Sn-containing perovskites.^13,24,46^

**Figure 1:**
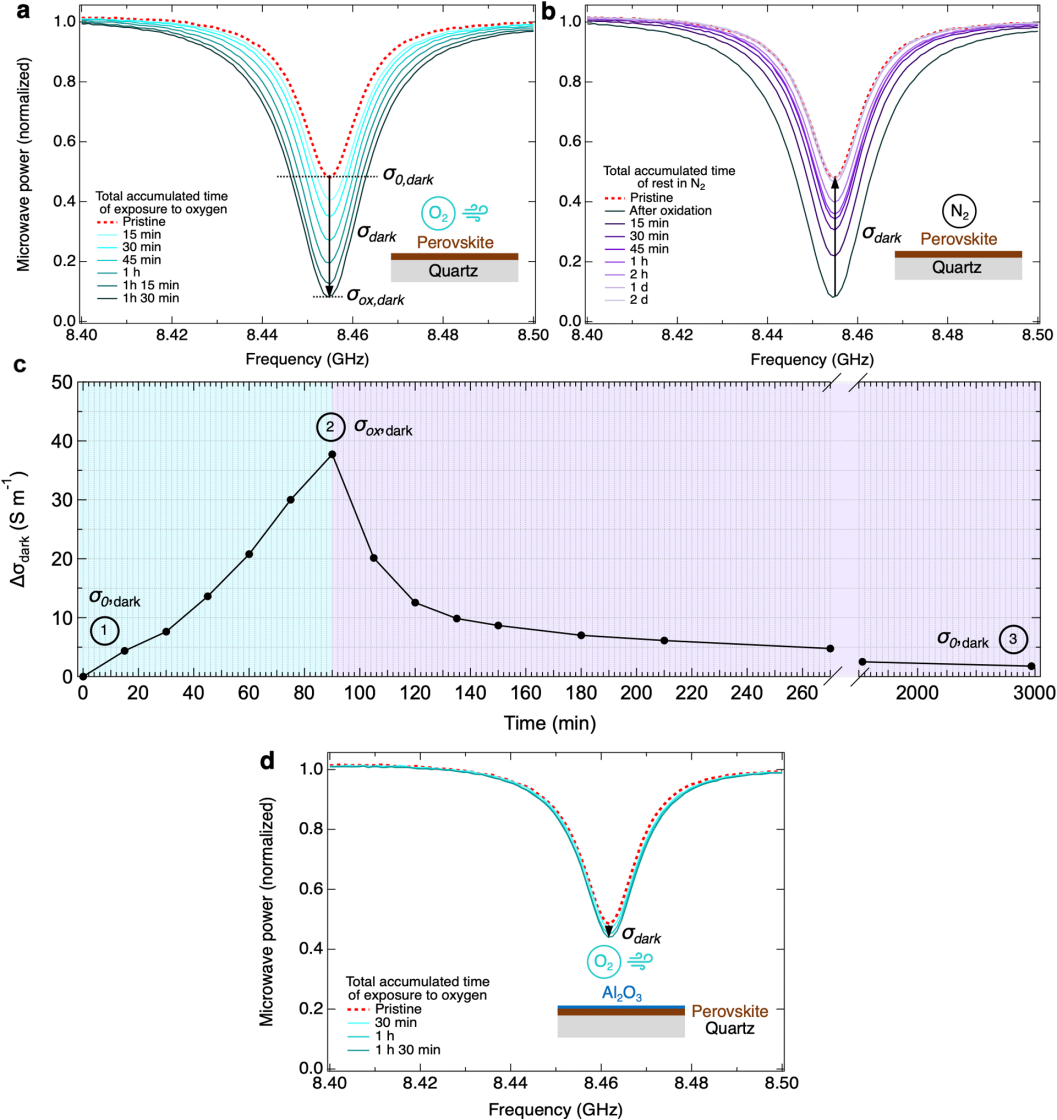
Frequency scans resulting from SSMC measurements of Sn_0.5_Pb_0.5_ perovskite thin films measured (a) upon exposure to oxygen and (b) after storage in N_2_. (c) Evolution of the dark conductivity (Δσ_dark_) showing a linear increase of σ_ox,dark_ upon exposure (light blue region) and its decay upon storage in N_2_ (purple region). (d) Frequency scans resulting from SSMC measurements of Sn_0.5_Pb_0.5_ layer with a thin alumina (Al_2_O_3_) protective layer deposited by 100 cycles atomic layer deposition (ALD) on top of the perovskite layer (in blue).

Then, we examined the effect of exposing the perovskite thin films to a dry 21% O_2_/79% N_2_ gas mixture by using a vacuum line, as presented in the Supporting Information E/M S5 (see Figure S5). The microwave cell was filled with the gas mixture and the conductivity of a Sn_0.5_Pb_0.5_ layer was measured as indicated by Scheme 1. From Figure 1a we can conclude that with increasingly longer periods of exposure to oxygen, a higher σ_dark_ is observed. After oxygen exposure, the microwave cell was filled with N_2_ gas and the conductivity was again measured after varying time intervals. Although the sample is not exposed to oxygen anymore, a significant background conductivity can still be observed. Interestingly, σ_dark_ decreases with time in N_2_, until the conductivity returns to its initial value σ_0,dark_ over days (see Figure 1b). We conclude that the background carriers disappear by consecutive oxidation reactions, which means that the perovskite thin film shows only a temporary increase in the background conductivity upon exposure to oxygen. We underline that for a pristine layer σ_dark_ does not change when stored in N_2_, as shown in Figure S9a. Therefore, it can be inferred that oxygen induces metastable doping in Sn_0.5_Pb_0.5_ perovskite. Figure 1c shows the temporal oxygen-induced change in dark conductivity, Δσ_dark_, over time from fitting the SSMC results of Figure 1a (light blue region in Figure 1c) and b (purple region in Figure 1c), calculated as detailed in the Supporting Information E/M S4. Interestingly, we see an approximate linear relationship between the increase in σ_dark_ and the accumulated exposure time to oxygen, which is very different from the exponential-like decrease of σ_dark_ back to σ_0_,_dark_ under storage in N_2_.

Hence, the reaction between the background free holes and perovskite appears to be so rapid that the carriers may immediately decay by consecutive reactions. Therefore, we suggest that the generation and decay of free holes occur at the same time during exposure to oxygen.

Interestingly, oxygen-induced doping can almost entirely be suppressed by covering the perovskite film with a thin Al_2_O_3_ encapsulation layer obtained by 100 cycles ALD as shown by Figure 1d. The ALD deposition process and parameters are described in detail in the Supporting Information E/M S3. Therefore, our results suggest that protecting the perovskite surface by depositing an encapsulation layer is a straightforward way to curb oxygen-induced doping prevalent in Sn-containing perovskites.

**Figure 2:**
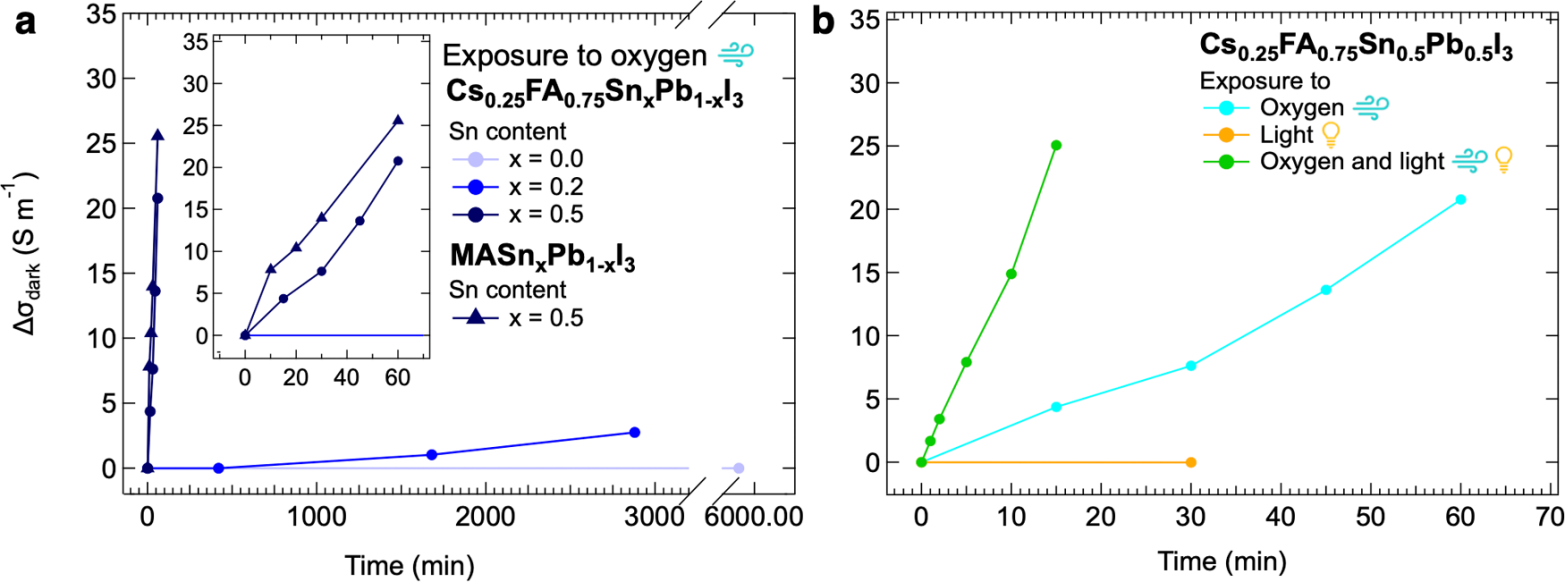
Change in dark conductivity (Δσ_dark_) over time for (a) ASn_*x*_Pb_1–*x*_I_3_ perovskite thin films with different A cations and different tin fractions *x* = 0.0, *x* = 0.2 and *x* = 0.5, where the inset shows the doping at shorter time scales, and (b) for Sn_0.5_Pb_0.5_ perovskite thin films upon exposure to oxygen (in light blue), light (in yellow) and simultaneously oxygen and light (in green).

To elucidate the underlying mechanism of oxygen-induced doping, we extended our research to thin films of perovskite with different tin fractions. As shown in Figure S10d, Sn_1_Pb_0_ exhibited a large σ_0,dark_ even before any exposure to oxygen, which means that the pristine layer is already substantially doped, disabling proper examination. For the Sn_0_Pb_1_ and Sn_0.2_Pb_0.8_ samples, the same procedure used to measure the Sn_0.5_Pb_0.5_ layer was used to obtain the Δσ_dark_ over time upon oxygen exposure. The results are shown in Figure 2a (see Figure S10a–c for the corresponding SSMC scans). For Sn_0_Pb_1,_ no increase in the conductivity is detected even after a total exposure time to oxygen of *t* = 4 d. For Sn_0.2_Pb_0.8_, we observe a rather small, but notable σ_ox,dark_ after *t* = 2 d, which is significantly less than the σ_ox,dark_ for Sn_0.5_Pb_0.5_, that is already visible after only *t* = 1 h. In line with our understanding, the increasing tin fraction leads to a higher susceptibility to oxygen-induced doping.

We also examined the effect of the cation at the A sites by measuring σ_dark_ for methylammonium mixed Sn–Pb perovskites (MASn_0.5_Pb_0.5_I_3_). The frequency scans in Figure S12a show that this sample also displays an increase in σ_dark_ after exposure to oxygen. By observing Figure 2a, we note that the susceptibility to oxygen-induced doping is similar to that of Cs_0.25_FA_0.75_Sn_0.5_Pb_0.5_I_3_. Therefore, we postulate that the doping process occurs irrespective of the choice of A-site cations.

To study how the oxygen-induced doping of Sn-containing perovskites is affected by light, after investigating the effect of exposure to oxygen only (see Figure S11a), we examined the effect of light only. Sn_0.5_Pb_0.5_ perovskite layers were placed in a N_2_-filled microwave cell and illuminated with a white LED, as described in the Supporting Information E/M S6. After a specific time interval, the LED was switched off and the perovskite layer was measured in the dark by SSMC. The frequency scans resulting from such SSMC measurements are visible in Figure S11b, while the corresponding evolution of Δσ_dark_ over time is shown in Figure 2b. We observed basically no increase in σ_dark_ upon illumination, implying that the dark conductivity of the perovskite thin films is not affected by light.

Next, the Sn_0.5_Pb_0.5_ perovskite films were exposed to oxygen and light simultaneously by introducing the 21% O_2_/79% N_2_ gas mixture into the microwave cell and illuminating by the white light LED with irradiance of 51 ± 1 mW cm^–2^. The frequency scans recorded in the dark are shown in Figure S11c, while the corresponding evolution of Δσ_dark_ over time is added to Figure 2b. We observe a rapid increase in background conductivity already after only *t* = 1 min. By comparing the rates of oxygen-induced doping, we notice an approximately 4 times increase in conversion rate by illumination. Hence, the combination of oxygen and light exposure accelerates the doping effect in Sn_0.5_Pb_0.5_ perovskite films. More specifically, we infer that oxygen induces doping and light enhances this process, since as mentioned before light alone does not lead to doping. Moreover, as shown in Figure S11c the conductivity decreased over days as the perovskite thin film is stored under N_2_, returning to its initial level σ_0,dark_, similarly to the samples which were exposed to oxygen only. Furthermore, as shown in Figure S12b, light-enhanced oxygen-induced doping also occurs in MASn_0.5_Pb_0.5_I_3_ thin films, although at an even faster rate compared to Cs_0.25_FA_0.75_Sn_0.5_Pb_0.5_I_3_. We conclude that the enhancement of oxygen-induced doping by light occurs independently on the choice of the A-site cation.

**Figure 3:**
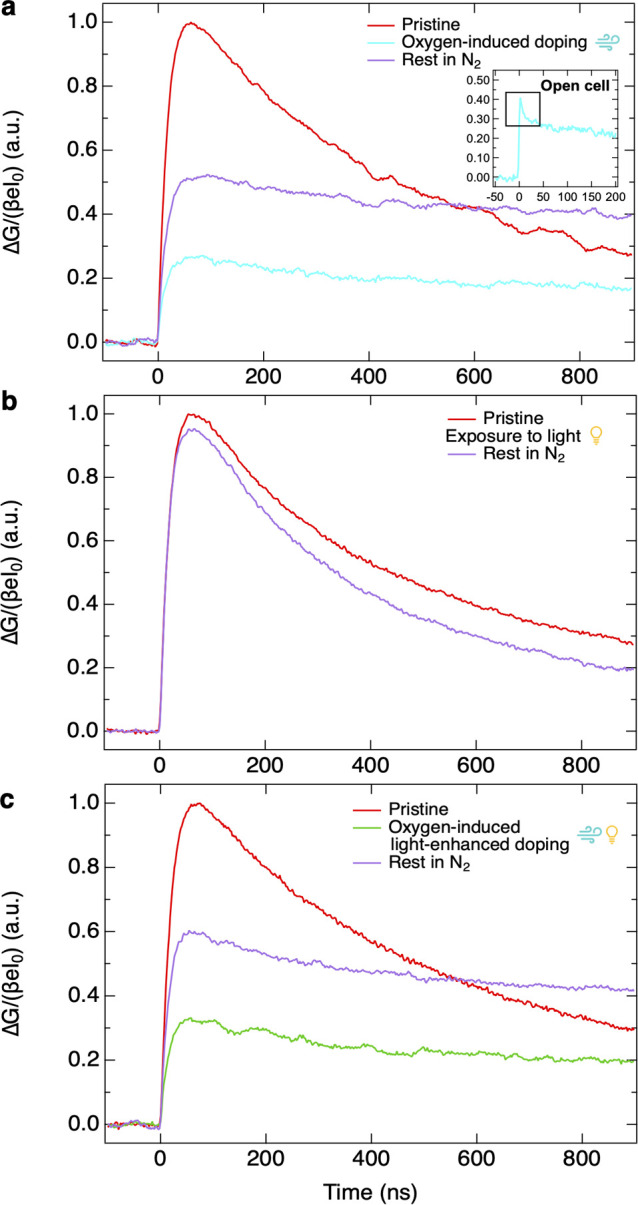
Effect of the exposure to (a) oxygen, (b) light and (c) simultaneously oxygen and light on the charge carrier dynamics of on Sn_0.5_Pb_0.5_ perovskite thin films measured by TRMC. The inset in (a) presents TRMC traces measured on shorter time scales (see also Figure S14). TRMC traces were recorded using identical laser intensities (∼(1–3) × 10^10^ photons cm^–2^) and wavelength (λ = 800 nm). All the TRMC traces have been normalized to the maximum signal of the corresponding pristine perovskite layer.

To improve the understanding of oxygen-induced doping and its enhancement by light, we investigated the charge carrier dynamics of Sn_0.5_Pb_0.5_ thin films by electrodeless laser pulse-induced TRMC, which is described in the Supporting Information E/M 4 and Figures S2 and S3. Note that all TRMC traces were measured after refilling the microwave cell with N_2_ to avoid that the laser light enhances the oxidation, as demonstrated in Figures S6 and S9b. To facilitate the comparison, the TRMC traces measured under pristine conditions, after exposure to oxygen, light or simultaneously oxygen and light and after storage in N_2_ were recorded using identical laser intensities and wavelengths. The results are shown in Figure 3. Additional TRMC traces recorded using various intensities are shown in Figure S13. To allow a comparison, all the TRMC traces have been normalized to the maximum signal of the corresponding pristine perovskite layer. The TRMC traces show a fast rise in the conductance due to the photogeneration of mobile charge carriers by the nanosecond laser pulse. The decay of the TRMC signal over time is due recombination or immobilization of the excess charge carriers in trap states. For the pristine films, the sum of electron and hole mobilities, Σμ, is in the order of 30–40 cm^2^ V^–1^ s^–1^ in combination with a photoconductivity signal lifetime exceeding τ > 1 μs. These mobility values are close to those reported in the literature obtained by optical-pump terahertz-probe measurements for similar perovskite compositions.^16,18^

Then, the TRMC traces recorded immediately after exposure to oxygen for *t* = 90 min are compared to those of the pristine layer (Figure 3a, respectively in light blue and red). We note that the doping induced by oxygen leads to a reduction of the TRMC signal to about one-third of the pristine film signal. We attribute this to fast pseudomonomolecular recombination of the photogenerated electrons with the background free holes, which reduces the charge carrier lifetime significantly. This is also evident by measuring the charge carrier dynamics by using a microwave open cell, which features a better time resolution, as visible in Figure S14. As shown in the inset of Figure 3a indeed, a rapidly decaying signal is visible. Hence, we suggest that the reduction of the TRMC signal is not so much related to a reduction in the charge carriers mobilities, but it is mainly the result of a rapid recombination induced by the high concentration of background free holes related to doping, leading to fast pseudomonomolecular recombination.

Apart from a reduction in signal size, the TRMC traces decay slower. As shown in Figure S15, long tails in the TRMC traces lasting for τ > 10 μs can be observed. These are indicative of trapping of one type of charge carrier, either electrons or holes, preventing recombination with their countercharges. Therefore, we conclude that apart from doping exposure to oxygen leads to the formation of additional defect states in the perovskite thin film. In support of defect formation, the reduction in photoconductivity and the increase in the first-order recombination rate were also observed in perovskite thin films with similar composition by transient THz photoconductivity measurements on exposure to ambient air.^18^ We emphasize that these defects are associated with oxygen-induced doping but are not necessarily directly responsible for the formation of free holes. It is more likely that they originate from consecutive oxidation products starting with the free holes.

As mentioned above, after oxygen exposure the dark conductivity decayed slowly to its original level σ_0,dark_ over a time scale of days when the films are stored in N_2_. However, the TRMC signal (Figure 3a, in purple) is only partially restored, since it is only half the signal of the pristine film. Note that these long-term effects do not occur when the similar layers are stored for days under N_2_ without any exposure to oxygen, as shown in Figure S9c–e. Hence, given that the oxygen-induced doping is almost entirely gone, rapid pseudo-first order recombination is not possible. This leads to a higher TRMC signal than that directly after oxygen-induced doping. Nevertheless, since we still observe a reduction in the TRMC signal with respect to that of the pristine sample in combination with the presence of the long-lived tails, we conclude that the defects are still present. Basically, this implies some irreversible deterioration of the perovskite layer, negatively affecting the charge carrier transport even after loss of doping has occurred.

Next, we checked the effect of illumination on the perovskite thin films in Figure 3b. On comparing the TRMC trace recorded after exposure to light for *t* = 30 min (Figure 3b, in purple) with the pristine film signal (Figure 3b, in red) we noticed that the dynamics of photogenerated charge carriers remain basically unchanged, except for a small variation in the size of the TRMC signal. Lastly, we investigated the effect on the charge carrier dynamics induced by the simultaneous exposure to oxygen and light. By comparing the charge carrier dynamics under pristine conditions (Figure 3c, in red), after the simultaneous exposure to oxygen and light for *t* = 15 min (Figure 3c, in green) and after storage in N_2_ for days (Figure 3c, in purple) to the dynamics shown in Figure 3a, we conclude that the TRMC traces exhibit the same changes as observed upon exposure to oxygen alone. This confirms that with light indeed the same oxygen-induced doping process occurs, creating the same type of defects as without light.

In the literature and as shown by eq 1, oxygen-induced doping is typically associated with p-type behavior.^13,31,32^ Hence, we postulate that the rise in conductivity is caused by an increase in mobile free holes formed on oxygen-induced doping. As detailed above from fitting of the SSMC measurements in Figure 1, Δσ_ox,dark_ is derived. From the TRMC measurements we obtain the charge carrier mobilities sum Σμ. Assuming μ_h_ ≈ μ_e_ on basis of the similar effective masses of the electrons and holes,^40^ we come to a hole mobility μ_h_ of around 15–20 cm^2^ V^–1^ s^–1^. By using eq 2, we can determine the actual concentration of mobile holes.
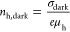
2

The initial charge carrier concentration for the pristine layers is *n*_0,h,dark_ ≤ 10^15^ cm^–3^. By comparing these values with the literature,^16,40,47^ we note that our perovskite thin films, especially those with higher tin fractions, are doped even in pristine conditions probably due to the inability of the SnF_2_ to prevent tin oxidation completely. The final doping level, *n*_h,dark_, is equal to the sum of the initial charge carrier concentration, *n*_0,h,dark_, and its increase due to doping, Δ*n*_h,dark_. After oxygen-induced doping, for Sn_0.5_Pb_0.5_ perovskite layers a moderate doping level of *n*_h,dark_ ∼ 7.0 × 10^16^ cm^–3^ is obtained after *t* = 1 h of exposure to oxygen. A similar doping can be accomplished in *t* = 13 min using simultaneously oxygen and light. For Sn_0.2_Pb_0.8_ a 5 times smaller free holes concentration is reached (*n*_h,dark_ ∼ 1.3 × 10^16^ cm^–3^) after *t* = 2 d of exposure to oxygen. For Sn_0_Pb_1_ the initial hole concentration is around *n*_h,dark_ ∼ 1 × 10^13^ cm^–3^ and does not change even after *t* = 4 d of exposure to oxygen.

Next, the potential impact of the oxidation on a corresponding perovskite solar cell is examined by studying the quasi-Fermi level splitting (QFLS), which is a measure of the maximum open circuit voltage attainable in a full device. We determined the QFLS by means of the microwave conductivity setup, as described in detail in Supporting Information E/M S7. Basically, we measure the photoconductivity on illuminating the perovskite layer using a green LED (λ = 522 nm) modulated at a frequency of 1 Hz at an intensity which generates the same amount of charge carriers as illumination under AM1.5. The change in voltage (Δ*V*) between light (light on) and dark (light off) over the microwave detector was probed by a lock-in amplifier and related to the conductivity. From the change in conductivity on illumination we calculate the excess charge carrier density, Δ*n*, using the known mobility. The QFLS can then be calculated by using eq 3.^48^
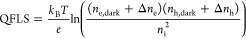
3where the *k*_B_*T*/*e* is the thermal voltage, *n*_i_ represents the intrinsic carrier density, *n*_e,dark_ and *n*_h,dark_ are respectively the dark electron and dark hole densities in thermal equilibrium, and Δ*n*_e_ and Δ*n*_h_ are respectively the photoinduced excess charge carrier densities.^48^

We calculated *n*_i_ ∼ 5 × 10^7^ cm^–3^ for mixed Sn–Pb perovskites by using the bandgap energy calculated in Figure S20 and the reported values of the effective masses.^40^ Since *n*_e,dark_ is expected to be close to 0 for a p-type doped semiconductor, the above expression reduces to eq 4.
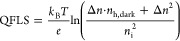
4

The QFLS results are collected in Table 1. On comparing the pristine film with the oxygen-treated samples, we observe an increase in the QFLS of almost 10 meV. This can be explained by realizing that on oxygen exposure we created metastable doping of the perovskite layer, which increases the Δ*n*·*n*_h,dark_ term in eq 4 substantially. This is independent if the perovskite film was exposed to only oxygen or simultaneously to oxygen and light, in agreement with our previous findings.

**Table 1:** Calculated QFLS Values in eV Measured by Means of the Microwave Conductivity Setup for Sn_0.5_Pb_0.5_ Perovskite Thin Films Under Pristine Conditions, After Oxygen-Induced Doping or Oxygen-Induced Light-Enhanced Doping and After Storage in N_2_

QFLS (eV)	pristine	after treatment	after storage in N_2_
oxygen-induced doping	0.97	1.07	1.00
oxygen-induced light-enhanced doping	0.97	1.05	1.01

Upon storage in N_2_, σ_dark_ decays slowly to its original level, reducing the Δ*n*·*n*_h,dark_ term again. The QFLS values decrease but remain slightly higher than those of the pristine films. We explain this by assuming that additional defects form due to consecutive oxidation products created by the free holes. We think that these defects are iodide vacancies, V_I_^•^, which give rise to electron traps. On optical excitation, excess electrons are readily trapped, yielding a long-lived excess holes concentration. With respect to the pristine layer, we anticipate that recombination of the trapped electrons to the ground state is very slow, leading to the observed increased QFLS values after storage in N_2_. This is in line with the slow recombination kinetics observed by TRMC in Figure 3c.

To get more insight into the oxidation mechanism, we analyzed the oxidation products formed on oxygen-induced (light-enhanced) doping. In the experiments described above where the Sn_0.5_Pb_0.5_ perovskite layers reach a moderate doping level of *n*_h,dark_ ∼ 7.0 × 10^16^ cm^–3^, after *t* = 1 h of exposure to oxygen. Since we know from eq 1 that each oxygen molecule reacting with tin leads to the creation of two free holes, only a very small fraction of the ions in the film is involved in the doping process. Indeed, if compared with the molar density of tin in the perovskite crystal structure (∼2.0 × 10^21^ cm^–3^), a fraction as small as ∼1.6 × 10^–5^ of the tin ions is converted to tin oxide species. This is in line with Figure S21, which shows that for Sn_0.5_Pb_0.5_ perovskite films the absorptance spectra do not change substantially. Moreover, we did not observe any significant changes (no disappearance of any perovskite XRD peak, change of crystal phase or new phases) in the corresponding XRD patterns, as shown in Figure S17. Taking this into account, we exposed the Sn_0.5_Pb_0.5_ perovskite layer to only oxygen or simultaneously oxygen and light for *t* ∼ 24 h, followed by a storage in N_2_ for *t* ∼ 7 days, to induce a substantial, structural change in the perovskite layer.

**Figure 4:**
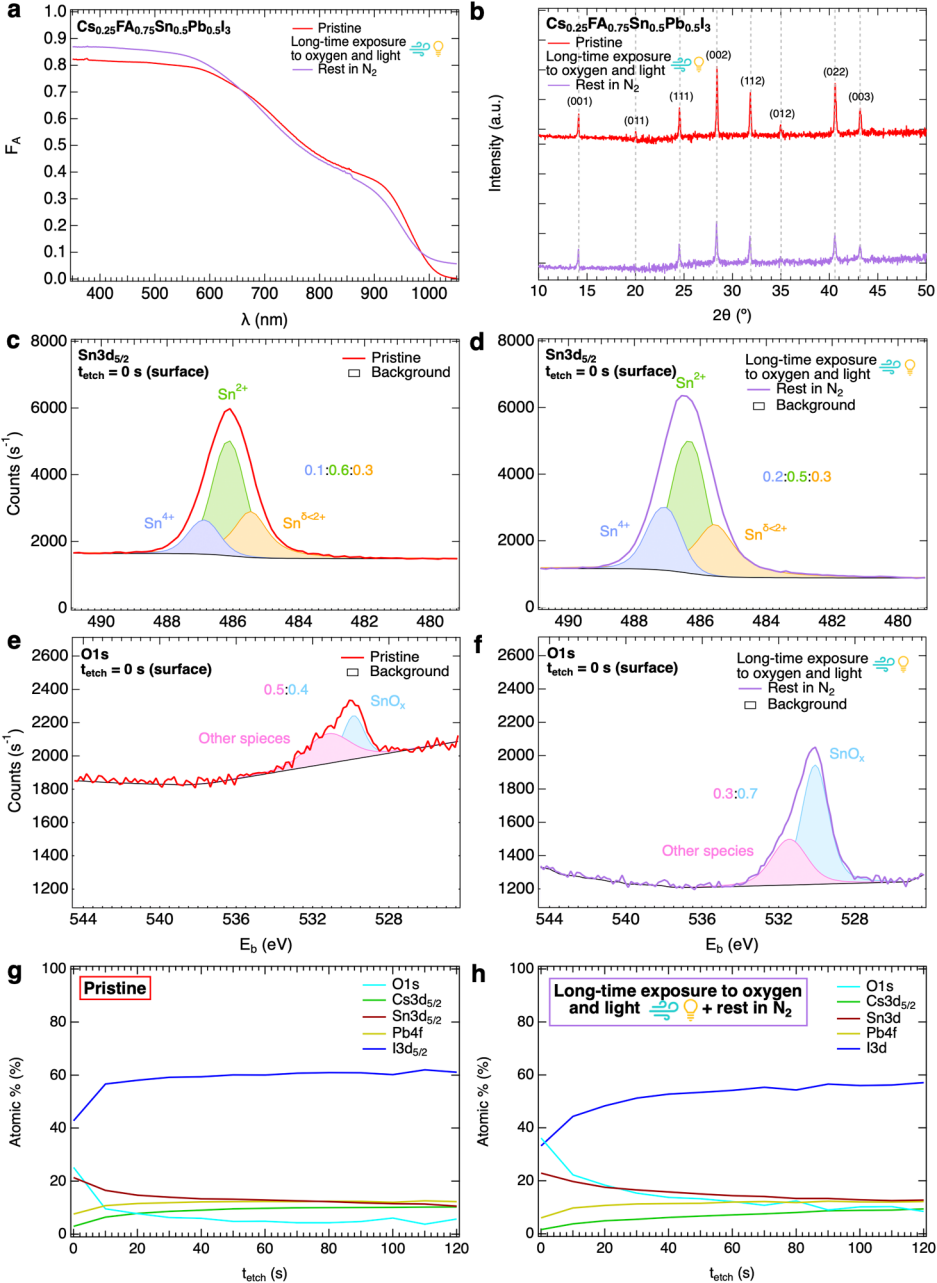
Comparison of optical, structural, and elemental properties of Sn_0.5_Pb_0.5_ thin films under pristine conditions (in red) and after exposure to simultaneously oxygen and light for *t* ∼ 24 h followed by storage in N_2_ (in purple). (a) Absorptance spectra and (b) XRD diffraction patterns, corrected with that of the quartz substrate, showing the Miller indices of the characteristic diffraction peaks of the pseudocubic crystal phase. (c–f) X-ray photoelectron spectroscopy (XPS) surface analysis and peak fitting presenting the elemental composition, oxidation states and their ratios for (c,d) Sn 3d_5/2_ and (e,f) O 1s core levels. (g,h) XPS depth profiling showing the relative percentages of O, Cs, Sn, Pb and I elements as a function of etching time (*t*_etch_), corresponding to a probing depth of about one-fourth of the total thickness of the film. The full XPS depth profiles are shown in Figure S23f,g.

After the prolonged treatment with simultaneously oxygen and light, we noted in Figure 4a a small reduction in absorption and a residual, enhanced optical absorption in the near-infrared spectral region. This is also visible for the prolonged treatment with only oxygen, as shown in Figure S22. Therefore, we suggest that prolonged treatments lead to the formation of optically active trap states within the bandgap. We underline that the optical changes are not related to free charges absorption since the dark conductivity has after the storage period reduced back to very low values. This defect formation is consistent with results of the TRMC measurements, however, the concentration of defects formed by short-term exposure is only marginal compared to that by long-term exposure.

On comparing the XRD patterns measured in vacuum of pristine and treated layers, as shown in Figure 4b for oxygen and light and Figure S18 for only oxygen, we observe a consistent lowering of all perovskite peaks after exposure, as also reported in the literature.^18,25,28,29,42^ We attribute this to a partial breakdown of the perovskite crystal structure. Moreover, we do not observe any new diffraction peaks, not even at the surface of the film as measured by grazing incidence X-ray diffraction and shown in Figure S19. Hence, the oxidation products may be amorphous, nanocrystalline or just a small fraction of SnO_*x*_ and/or volatile products such as I_2_.^18,25,27−29,42,49^

Next both treated and pristine films were analyzed using XPS as shown in Figure S23 and Table S1. By fitting the peaks of the Sn 3d_5/2_ and O 1s core levels at the surface presented in Figure 4c,e, we observe even for the pristine film tin oxide species (SnO_*x*_) at the surface. This tin oxidation is probably due to undesired residual oxygen in the N_2_-filled glovebox or by the DMSO solvent used in the perovskite synthesis, as reported in the literature.^50,51^ Nevertheless, as shown by Figure 4d,f, long-term exposure to simultaneously oxygen and light leads to further oxidation of tin from Sn^2+^ to Sn^4+^ and formation of additional SnO_*x*_ as oxidation product. In fact, ignoring the residual adsorbed oxygen on the surface of the films and comparing the regions just below the surface (*t*_etch_ = 10 s) in Table S2, we notice that the Sn/O ratio is higher for the treated (1:1.1) than for the pristine (1:0.6) layer, which confirms oxygen incorporation and the formation of SnO_*x*_ near the surface. Furthermore, the XPS peak associated with other oxide species is most likely due to C=O bonds between adventitious carbon and residual oxygen. In addition to that, Figures 4c,d reveal that undercoordinated tin ions Sn^δ<2+^ (where δ is the oxidation state), observed in the literature as well,^27^ are also present at the film surface for both pristine and treated layers. We attributed this to I-poor conditions caused by the addition of SnF_2_ as an additive or a lack of incorporation of iodide during the perovskite synthesis. In line with this, a subtle depletion of iodide at the pristine film surface is observed in the XPS depth profiling in Figure 4g and the Sn/I ratios in Table S2, where the Sn/I ratio is only 1:2 instead of 1:5.8 deeper in the bulk of the film (closer to the expected 1:6 ratio). The lack of iodide becomes evident after the long-term exposure, as shown in the XPS depth profiling in Figure 4h. In this case, the Sn/I ratio are 1:1.5 at the surface and 1:4.5 deeper into the film thickness. Similarly, we observed tin oxidation, additional SnO_*x*_ formation and iodide depletion by XPS analysis of the perovskite layer surface after exposure to oxygen for *t* ∼ 24 h and rest in N_2_ for *t* ∼ 7 d, shown in Figure S24 and Table S3. However, it seems that the prolonged exposure to only oxygen caused also a slight shift to higher binding energies (*E*_b_) of the XPS peaks of lead and iodide at the surface, the latter being in line with the literature and associated with the formation of I_3_^–^.^27^ This suggests a variation in the chemical bonds involving these ions, which we believe to be due to a slight oxidation and thus degradation of the perovskite crystal structure.

From the XPS results we infer the formation of SnO_*x*_ and partial volatilization of I_2_. During the storage in N_2_, background holes are expected to oxidize iodide to iodine, leading to the evolution of I_2_,^29^ as shown by eq 5, expressed by Kröger-Vink notation.
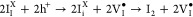
5

We suggest that iodide Frenkel defects form, which are reported as an iodine-related degradation pathway for other perovskites.^52,53^ In detail, in the process shown in eq 5 two iodide ions, I_I_^X^, with neutral charge since they occupy the right lattice sites in the perovskite crystal, are oxidized by background holes and displaced from their positions to occupy interstitial sites. Then, two oxidized interstitial iodide ions, I_i_^X^, which form neutrally charged defects in the lattice, are converted into I_2_. The evolution of I_2_, is thus accompanied by the formation of two positively charged iodide vacancies, V_I_^•^. This is in line with our hypothesis of formation of V_I_^•^ crystal defects on interpretating the QFLS results in Table 1.

The formation of I_2_ in Sn-containing perovskites in the presence of oxygen, with or without simultaneous illumination, has also been observed in the literature.^25,27,28,49^ This reaction leads to undoping of the system, explaining the metastability of the oxygen-induced (light-enhanced) doping, and depletion of iodide from the film surface. This is in line with the weaker bonds between tin and iodide in the perovskite crystal structure upon tin oxidation discussed in the literature,^19^ and with the above observations on the perovskite degradation.

**Scheme 2:**
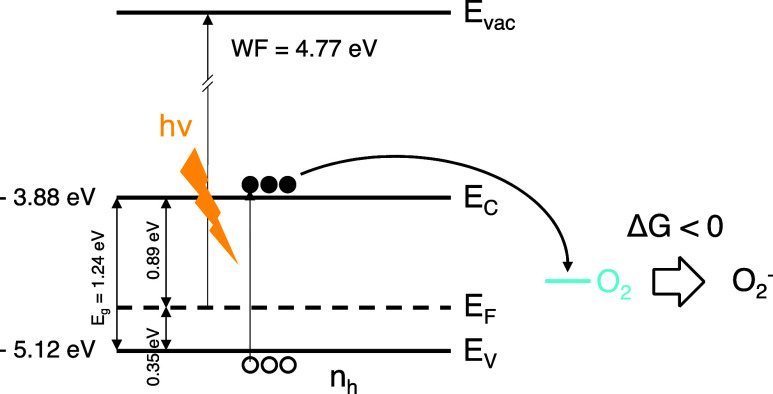
Illustration of the Process of Light Enhancement of Oxygen-Induced Doping in ASn_0.5_Pb_0.5_I_3_ Perovskite Films, where *E*_vac_ = Vacuum Level, *E*_C_ = Conduction Band Minimum, *E*_V_ = Valence Band Maximum, *E*_g_ = Bandgap Energy, Δ*G* = Change in Free Energy of the Reaction Between the Initial and Final States and *n*_h_ = Background Free Holes Concentration, Here Considered to be *n*_h,dark_ ∼ 10^16^–10^17^ cm^–3^. This Illustration is Partially Based on Values Reported in the Literature for Similar Perovskites^21,47^

Lastly, we try to address the enhancement observed on oxygen-induced doping under illumination. Although there is a huge excess of oxygen in the microwave cell, the oxidation reaction without light is sluggish.^29,54^ Hence, we conjecture a reaction-limited mechanism depending on the probability of interaction between oxygen and tin. In the case of light-enhanced oxygen-induced doping, electrons are excited to the conduction band.^28^ We hypothesize that these conduction band electrons react with O_2_ forming superoxides (O_2_^–^) as an intermediate reaction product, which is also reported in the literature.^28,54,55^ It is conceivable that the free energy for these conduction band electrons to react with oxygen is substantially favored in comparison to valence band electrons, explaining the enhanced conversion rate by light. A schematic illustration of this process is shown in Scheme 2. Furthermore, we suppose that under illumination part of the iodide ions becomes mobile interstitial lattice defects,^27^ due to the low activation energy for iodide migration (0.3–0.5 eV),^30^ favoring the degradation of the perovskite layer.

Understanding the mechanism underlying light-enhanced oxygen-induced doping is extremely important, as we showed that it impairs the charge carrier dynamics in mixed Sn–Pb perovskites, limiting ultimately the device efficiency. Our results point out that future research should focus on finding solutions to prevent the reaction of the perovskite absorber with oxygen. The presence of oxygen during the material synthesis and the fabrication of the solar cell or a small leak in the full device encapsulation will be detrimental. This is particularly worrying when one considers that harvesting solar energy relies precisely on exposure of the absorber layer to sunlight. Apart from preventing the presence of oxygen during the entire manufacturing process and the correct selection of durable encapsulation materials, a combination of additives and surface passivation, e.g. via a thin Al_2_O_3_ ALD layer, may be a successful strategy to suppress oxygen-induced doping. We envision that this plan of action could lead to the implementation of highly efficient and long-term stable devices in the coming years.

## Conclusions

In this work, we investigated qualitatively and quantitatively oxygen-induced doping in isolated mixed Sn–Pb perovskite thin films. For Cs_0.25_FA_0.75_Sn_0.5_Pb_0.5_I_3_ incrementally longer exposure times to oxygen leads to a progressively higher dark conductivity, which decays exponential-like to its original level over a time scale of days when the films are stored under N_2_, revealing a metastable process. The photoconductivity, however, does not revert to its original kinetics, implying a permanent degradation of the charge transport properties. Additionally, oxygen-induced doping is accelerated by illuminating the perovskite. We observed that oxygen-induced doping is unique for the tin-containing perovskites and occurs irrespective of the choice of A cation(s). We conclude that the exposure to oxygen leads to the oxidation of tin to SnO_*x*_ species, which entails the creation of free holes which effectively p-dope the perovskite, and formation of additional defect states, which both enhance the recombination of photogenerated carriers. In contrast to the relatively small doping level, this is sufficient to cause immediate and enormous changes in the charge carrier dynamics. Prolonged exposure of the mixed Sn–Pb perovskite films to oxygen and light is required to reveal measurable structural and optical changes in the perovskite film. On oxidation, the perovskite crystal structure deteriorates due to a buildup of tin oxide species, SnO_*x*_, and loss of iodide due to the release of I_2_ near the surface. Basically, we state that the defect density arising from short-term exposure to oxygen immediately impairs the solar cell optoelectronic properties, while perovskite structural and optical properties degradation only emerges upon long-term exposure and accumulation of oxidation products. We believe that the exposure to oxygen of mixed Sn–Pb perovskites solar cells during production and operation should be strictly prevented to improve their performance and lifetime. Moreover, understanding the oxygen-induced degradation processes is of fundamental importance to select the best materials, device architectures and encapsulation, and appropriate fabrication conditions.

## Supporting Information Available

Experimental section/Methods, SSMC and TRMC results, quantification of tin oxidation, XRD, UV–vis–NIR spectroscopy and XPS results, Figures S1–S24, Tables S1–S3 (PDF)
