# Supplementary material for: Metastable Oxygen-Induced Light-Enhanced Doping in Mixed Sn–Pb Halide Perovskites
Source: J Am Chem Soc. 2024 Nov 4;146(45):30860–70. doi: 10.1021/jacs.4c08924 (PMC11565635; doi:10.1021/jacs.4c08924)
Supplement: Supplementary file 1 — ja4c08924_si_001.pdf [file ja4c08924_si_001.pdf]

Metastable Oxygen-Induced Light-Enhanced Doping in Mixed Sn-Pb Halide Perovskites

Jasmeen Nespoli[†], Matthijs Mugge[†], Lara M. van der Poll[†], Snigdha Lal[†], Bahiya Ibrahim[†], Bart Boshuizen[†], Valentina M. Caselli[†], Arjan J. Houtepen[†], Lars J. Bannenberg[‡], and Tom J. Savenije[*,†]

[†]*Department of Chemical Engineering, Faculty of Applied Sciences, Delft University of Technology, 2629 HZ Delft, The Netherlands*

[‡]*Department of Radiation Science and Technology, Faculty of Applied Sciences, Delft University of Technology, 2629 JB Delft, The Netherlands*

[*] E-mail: T.J.Savenije@tudelft.nl

Supporting Information (SI)

Experimental section/Methods (E/M)

E/M 1) Materials

All materials were used as received. Cesium iodide (CsI, 99.999%) and tin (II) fluoride (SnF_2, 99%) were purchased from Merck-Sigma Aldrich**.** The organic halide salt formamidinium (FAI, 99.99%) was purchased from Greatcell Solar Materials. Lead (II) iodide (PbI_2, 99%) was purchased from Acros Organics and tin (II) iodide (SnI_2, 99.999%, mesh beads) was purchased from Alfa Aesar. Dimethylformamide (DMF, anhydrous, 99.8%), dimethyl sulfoxide (DMSO, anhydrous, ≥ 99.9%) and anisole (anhydrous, 99.7%) were purchased from Merck-Sigma Aldrich.

E/M 2) Mixed Sn-Pb perovskite thin film production

Prior to the deposition of the mixed Sn-Pb perovskites thin films, the quartz substrates were prepared by performing an ultrasonic bath for 5 min in acetone and 5 min in isopropanol. The substrates were then left to dry completely and rubbed with an antistatic wipe to remove any residue before an UV-ozone cleaning treatment lasting 10 min. All the steps relative to the perovskites thin films production were carried out in a glovebox with low levels of $O_2 \lesssim 0.5$ ppm and $H_2O \simeq 0.8$ ppm. Two parent solutions of pure Pb-based perovskite with composition $Cs_{0.25}FA_{0.75}PbI_3$ and pure Sn-based perovskite with composition $Cs_{0.25}FA_{0.75}SnI_3$ (1.5 M) were prepared by dissolving and stirring overnight the specific perovskite precursors (CsI/FAI/PbI_2 in molar ratio 1:3:4 for the pure Pb-based perovskite and CsI/FAI/SnI_2 in molar ratio 1:3:4 for the pure Sn-based perovskite) in a solution of DMF and DMSO with a volumetric ratio of 4:1.

SnF_2 (20mol% relative to SnI_2) was added to the tin precursor solution. The different solutions to obtain $Cs_{0.25}FA_{0.75}Sn_xPb_{1-x}I_3$ perovskites with variable tin content, indicated hereafter as Sn_xPb_{1-x}, were prepared by mixing the two parent solutions in appropriate volume ratios and subsequently stirred for 1 h. Then, the mixed Sn-Pb perovskites polycrystalline thin films were prepared by antisolvent spin-coating. The solutions were dripped evenly onto the substrate and spin coated with a rotational speed ramp of 500 rpm and a final speed of 3000 rpm for 60 s. After 50 s from the beginning of the rotation, 200 μL of anisole (antisolvent) were poured gently but firmly in ≤ 1 s from approximately 1-1.5 cm above the surface of the sample to initiate a flash reaction resulting in the crystallization of the perovskite thin film. Lastly, an annealing at 100 ºC for 10 min is performed immediately afterwards. The final thickness of the perovskite thin films is ~ 250 nm on average.

E/M 3) Atomic layer deposition

An ultrathin passivation layer of alumina (Al_2O_3) was deposited by atomic layer deposition (ALD) on the surface of the perovskite thin films by using a Veeco Fiji G2 ALD reactor. The transfer of the perovskite layers in the load lock before the reactor chamber was carried out as fast as possible in order to limit the exposure to ambient air to < 30 s. Thermal ALD was carried out at a temperature of 100ºC under high vacuum conditions ($P = 10^{-7}$ torr). The process was based on the sequential deposition of H_2O and TMA (trimethylaluminium, $Al_2(CH_3)_6$) as precursors, starting with introducing H_2O vapor in the reactor chamber, which created a single layer by being absorbed and reacting on the surface of the samples, followed by TMA vapor. The reactor chamber was purged with N_2 after each step to remove all residual precursors and reaction products. To obtain an encapsulation layer, 100 alternating cycles ALD were performed. The thickness of the Al_2O_3 ALD layer was estimated to be ~ 10 nm assuming a deposition rate of 1 Å/cycle of alumina on perovskite.

E/M 4) Steady-state microwave conductance and time-resolved microwave conductivity

The charge carrier properties in the perovskite thin films before and after oxygen exposure (or after simultaneous oxygen and white light LED exposure) was investigated by microwave-based characterization techniques.

The steady-state microwave conductance (SSMC) technique was used to inspect the background conductivity in the dark, σ_{dark}, of perovskite thin films, i.e. the doping level. The interaction between perovskite and ambient air was prevented as each sample under investigation was located in a specific microwave cell sealed inside a N_2-filled glovebox. In this way, the initial background conductivity, $\sigma_{0,dark}$, was obtained. For the oxygen exposure experiments, the microwave cell was filled with O_2 for a certain period of time, the background conductivity after exposure to oxygen, $\sigma_{ox,dark}$, was obtained, and then the cell was filled with N_2 again. On the other hand, for the oxygen and light simultaneous exposure experiments, the microwave cell was filled with O_2 and white light was shone on the sample at the same time by using a LED lamp. Then, after a certain elapsed time, the lamp was switched off and the background conductivity after simultaneous exposure to oxygen and light, $\sigma_{ox+light,dark}$, was obtained. Then, the

microwave cell was filled with N_2 again. Hence, all SSMC measurements were performed in the dark, with a different gas filling the cell depending on the experiment. The microwaves source (frequencies between 8.2-12.2 GHz) is a voltage-controlled oscillator. The microwaves pass through the perovskite thin film located in the microwave cell and they are partially absorbed due to the interaction with free, mobile charge carriers. The type of microwave cells used for the SSMC measurements is a cavity cell, which allows the creation of a standing wave at a specific resonant microwave frequency by partially closing the cell with an iris. The standing wave passes multiple times throughout the sample. The resulting high-sensitivity measurement enables charge carriers in the perovskite layer to be excited at a laser intensity as low as 10^8 photons cm^{-2}, although the drawback of a delay in the response time of 18 ns. A circulator separates the incident from the reflected microwaves, which are recorded by a microwave detector and a signal processing system in the form of a reduction in the microwave power (ΔP) between the reflected and the incident microwave.[1] During a SSMC measurement, the microwave power loss is probed while sweeping across the microwaves frequency range. As schematically illustrated in **Figure S1a**, at the resonant microwave frequency of the system made of the perovskite thin film deposited on quartz under investigation and the cavity cell, a standing wave forms inside the cavity. The maximum of the microwave standing wave overlaps the sample at approximately ¾ of the cell length, resulting in a highly sensitive measurement of the maximum microwave power loss. This emerges as a dominant dip at the resonant frequency in the microwave frequency scan, as it can be observed in **Figure S1b** for a quartz substrate and a perovskite thin film deposited on the same quartz substrate.[1,2]

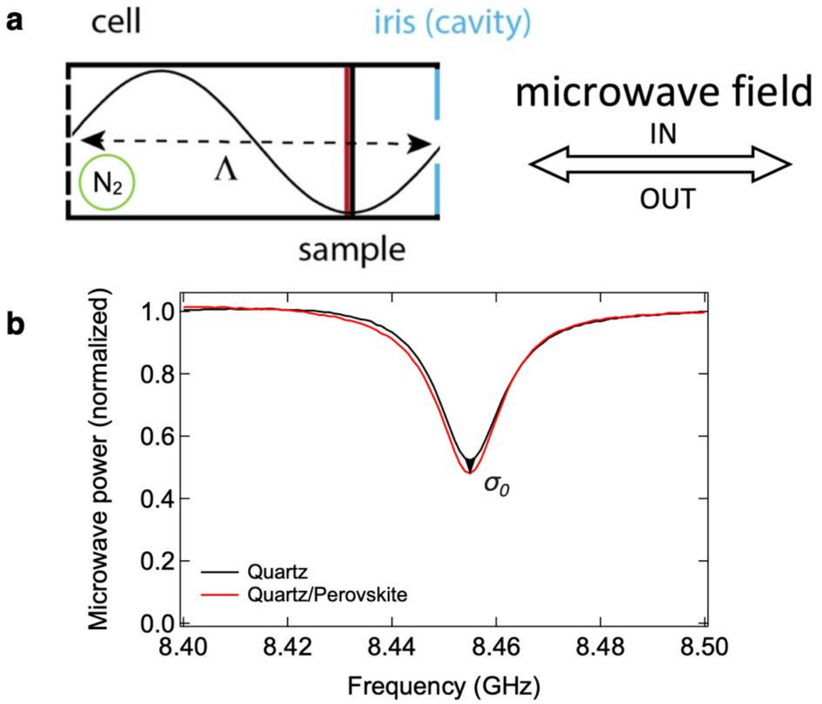

Figure S1: SSMC setup and measurement. (a) Illustration representing the interaction between the microwaves and sample under investigation in the fully reflective cavity cell of length Λ. The microwave field takes the form of a stationary wave of wavelength Λ at a specific resonant frequency.[1] (b) Frequency scans measured by SSMC, showing

the results for a quartz substrate and a perovskite thin film deposited on it. The frequency scan of quartz is used as a reference, since its electrical insulator characteristics define the lower limit of detection in measuring the background conductivity. The resonant frequency dips can be modelled and by comparing the dips of the quartz substrate and of the perovskite thin film deposited on it, which is a conductive film leading to further dip deepening, σ_0 of the perovskite layer can be obtained.

All the SSMC measurements are normalized to a frequency scan of the fully reflective endplate of the cavity cell. As shown in **Equation (S1)**,[2] the normalized maximum change in microwave power can be related to the total variation in the conductance (ΔG) of the perovskite thin film, which corresponds to the integrated change in electrical conductivity ($\Delta\sigma$) over the full perovskite layer thickness.

$$\frac{\Delta P}{P} = \frac{P' - P}{P} = -K\Delta G = -K\Delta\sigma\beta L \qquad (1)$$

Where K is the sensitivity factor of the microwave cell, β is the ratio between the inner walls of the microwave guide of the microwave cell and L is the perovskite thin film thickness.[2]

The change in conductivity $\Delta\sigma$ measured by SSMC technique derives from the change in background charge carrier concentration, Δn, in equilibrium, in the dark at room temperature. Hence, the normalized microwave power loss signal obtained by SSMC can be fitted to estimate the σ_{dark} of the sample and then calculate the background carrier density, n_{dark}, by using **Equation (S2)**.[1]

$$\sigma_{dark} = e\sum \mu n_{dark} = e(\mu_e n_{e,dark} + \mu_h n_{h,dark}) \qquad (2)$$

Where e is the elementary charge and $\mu_{e/h}$ *and* $n_{e/h}$ are, respectively, the mobilities and concentrations of the background carriers.[1] **Equation (S2)** can be used to derive the initial background charge carrier concentration $n_{0,dark}$.

The frequency scan of the perovskite layer is compared to that of a bare, identical quartz substrate (see **Figure S1b**). Basically, when the frequency scans of the perovskite thin film and quartz are almost the same, we deduce that σ_{dark} is relatively low. On the other hand, in case of doping the corresponding σ_{dark} would lead to enhanced microwave absorption, which results in a deepening of the resonance frequency dip.

In case of perovskite doping, the following **Equation (S3)** is applied.

$$\sigma_{dark} = e\mu n_{dark} \qquad (3)$$

In this case, the background conductivity mainly results from the contribution of only one type of charge carrier and only its mobility needs to be taken into account. For instance, the change in background hole concentration $\Delta n_{h,dark}$ in the case of p-type doping can be calculated using **Equation (S4)**.

$$\Delta n_{h,dark} = \frac{\Delta\sigma_{dark}}{e\mu_h} \tag{4}$$

Where only the mobility of holes, i.e. the majority carrier, is taken into account. When the effective masses of electrons and holes are similar, it is possible to assume that both present the same mobility and thus $\mu_{e/h} = \mu/2$.

Besides, the time-resolved microwave conductivity (TRMC) technique was used to study the charge carriers dynamics and transport properties in perovskites thin films.

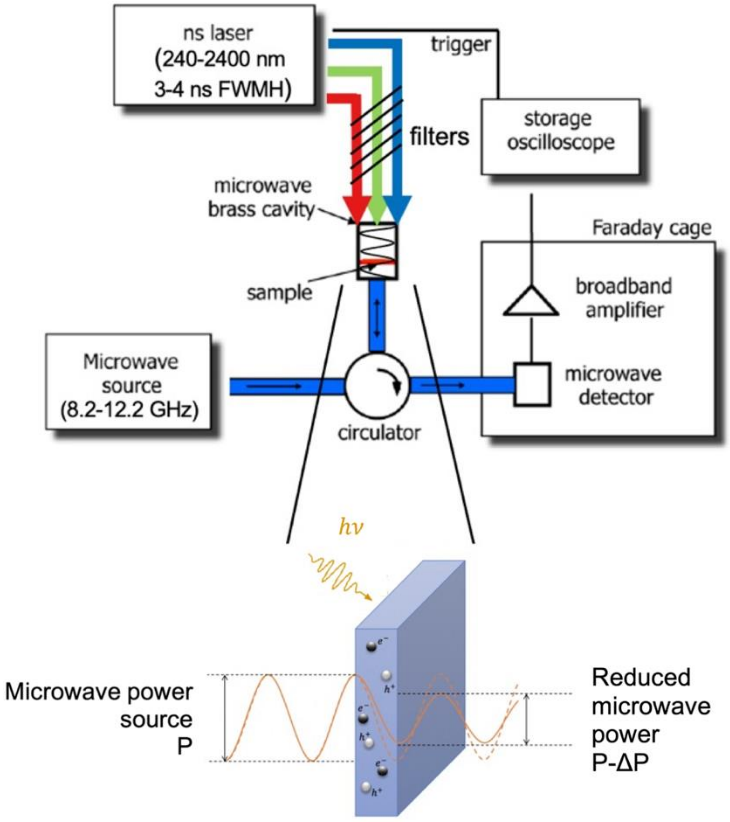

Figure S2: Schematic illustration of the TRMC setup, showing the interaction between the oscillating microwave electric field and the sample under investigation in more detail.[1]

The same working principle and set up based on the use of the microwave cavity cell described above for the SSMC technique applies to the TRMC technique. The main difference is that the TRMC setup, schematically illustrated in **Figure S2**,[1] presents an ultrafast Nd:YAG laser which is used to generate pulses of the duration of ~ 3.5 ns on average

at a repetition of 10 Hz. The laser pulses make electrons to be excited to the conduction band, leaving holes in the valence band. In all TRMC measurements, the light intensity of the excitation laser pulse was tuned between 10^8 and 10^{11} photons cm^{-2} by using an array of neutral density filters. During a TRMC measurement, the reduction of the microwave power resulting from the interaction between the microwave standing wave forming inside the cavity cell and the photogenerated free carriers is recorded as a function of the time elapsed after the laser pulse ($\Delta P(t)$). The normalized reduction in microwave power is related to the time by the time-dependent form of **Equation (S1)**.[1]

In addition to the microwave cavity cell, it is also possible to use a microwave open cell without an iris. In this case, the microwave passes only one time throughout the sample. As a result, the instrumental response time is reduced to 2 ns compared to 18 ns for the cavity cell, but this is at the expense of a loss of sensitivity with a 10-fold lower K factor than the cavity cell which requires increasing the laser intensity by two orders of magnitude typically up to 10^{13} photons cm^{-2}.

By knowing the specific sensitivity factor and the measured microwave power loss as a function of time, the time-resolved change in photoconductance between dark and after illumination can be quantitatively calculated ($\Delta G(t)$). Similarly to **Equation (S2)**,[1] the time-dependent variation in photoconductance is related to the time-dependent variation in electrical conductivity ($\Delta\sigma(t)$), which scales with the time-dependent concentration ($n_e(t)$ and $n_h(t)$, namely the lifetime relative to the recombination rate), and mobilities sum ($\mu = \mu_e + \mu_h$) of free electrons and holes, as shown in **Equation (S5)**.[1]

$$\sigma(t) = e \sum \mu n(t) = e(\mu_e n_e(t) + \mu_h n_h(t)) \tag{5}$$

In order to directly compare different samples, the maximum TRMC signal can be expressed by the product of charge carrier yield and gigahertz-frequency mobilities sum. If every absorbed photon generates a single electron-hole pair, which commonly occurs in direct bandgap perovskites with low exciton binding energy at room temperature, the yield of free charge carrier generation (φ) is equal to 1. This is expressed by **Equation (S6)**.[1]

$$\phi = \frac{Ln}{F_A I_0} \tag{6}$$

Where I_0 is the intensity of the laser (photons per laser pulse per unit area) and F_A is the absorbed fraction of light at the excitation wavelength. By combining **Equation (S2)** and **Equation (S6)**, the equation relating ΔG_{max}, the charge carrier yield and mobilities sum can be obtained, as shown in **Equation (S7)**.[1]

$$\phi(\mu_e + \mu_h) = \frac{Ln}{F_A I_0}\frac{\Delta\sigma}{en} = \frac{L}{F_A I_0}\frac{\Delta G_{max}}{e\beta L} = \frac{\Delta G_{max}}{F_A I_0 \beta e} \tag{7}$$

The relationship between these important properties can be seen in the transient photoconductance signals, referred to as TRMC traces, in **Figure S3**.

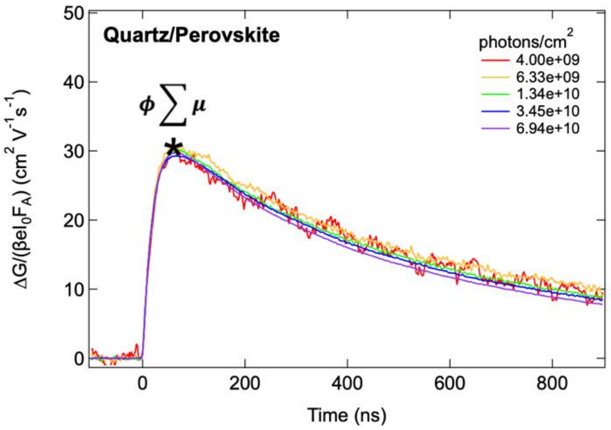

Figure S3: Visualization of the relationship between the maximum variation of photoconductance signal ($\Delta G_{max}/(\beta e I_0 F_A)$) and the product between the free charge carrier generation yield and the mobilities sum ($\varphi\Sigma\mu$) in a typical transient photoconductance signal obtained by TRMC.

Lastly, all TRMC measurements were corrected for the appropriate sensitivity factor of the microwave cavity cell for the mixed Sn-Pb perovskites thin films under investigation. Since the sensitivity factor increases as the background conductivity increases due to the different interaction between microwaves and background charge carriers that modifies the TRMC signal, the influence of the level of doping on the sensitivity factor was taken into account. The variation in K factor as a function of σ_{dark} for mixed Sn-Pb perovskite thin films of different composition and various doping levels were calculated by fitting the frequency scan dips by using a custom-built computer program which solves the Maxwell equations in the resonant cavity by considering the resonance phenomenon and the dimensions of the cavity cell, as well as the dielectric properties of the layers located into it. The feasibility of the fitting depends on the minimum and maximum level of detection of, respectively, $\sigma_{0,dark,min}$ = 10^{-3} S m^{-1} and $\sigma_{ox,dark,max}$ ~ 85 S m^{-1}. The first is determined by the intrinsic conductivity of quartz, while the latter depends on the magnitude of the microwave perturbation, which in case of heavily doped samples can become very strong and lead to unreliable estimations. As a result, it was calculated that K ~ 66,000 for perovskite layers in pristine conditions and increases with increasing background conductivity (doping) with a nonlinear relationship, reaching K ~ 92,000 for $\sigma_{ox,dark}$ ~ 40 S m^{-1}. As an example, **Figure S4** shows the variation of the K factor of the microwave cavity cell as a function of the background conductivity for a perovskite thin film of composition $Sn_{0.5}Pb_{0.5}$. For the TRMC measurements performed by using the open microwave cell, no relevant adjustment of the K factor according to the background conductivity was necessary, considering its 10-fold lower sensitivity compared to the cavity cell. Hence, the K factor was kept equal to K = 1,000.

However, calculations to determine the change in the sensitivity factor were proven unsuccessful in the case of either substantially low/high $\sigma_{0,dark}/\sigma_{ox,dark}$ when the lower/upper limit of detection of the TRMC was reached. Nevertheless, in these cases, the most accurate but approximate value of sensitivity factor was used for the correction.

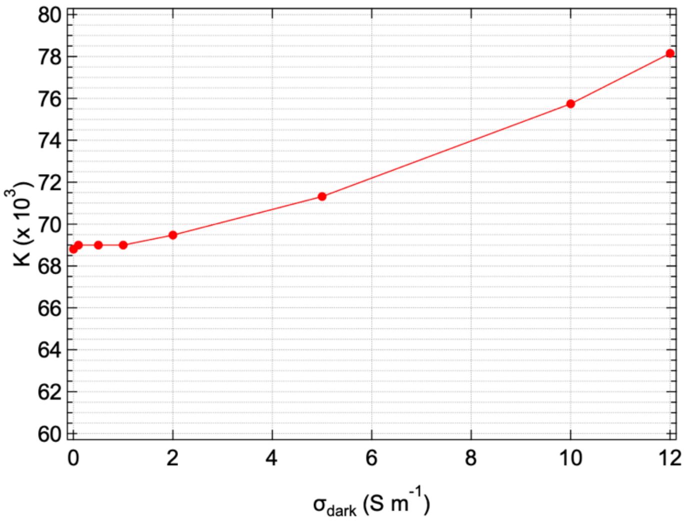

Figure S4: Calculated variation of the *K* factor of the microwave cavity cell for mixed Sn-Pb perovskite thin films of composition $Sn_{0.5}Pb_{0.5}$ as a function of the background conductivity (doping) in the range σ_{dark} = 0-12 S m^{-1}.

E/M 5) Vacuum line setup for oxidation of mixed Sn-Pb perovskite thin films

The oxidation of mixed Sn-Pb perovskite thin films was carried out via a vacuum line connected to a specific microwave cavity gas cell containing the sample under investigation, a vacuum pump for pre-pumping, a turbo-pump for high vacuum pumping, a bottle of 21% O_2 / 79% N_2 gas mixture and a bottle of high-purity N6.0 N_2 gas. The schematic illustration of the oxidation setup is shown in **Figure S5**. The perovskite thin film under investigation was located in the microwave cavity gas cell sealed inside a N_2-filled glovebox with low levels of $O_2 \lesssim 1$ ppm and $H_2O < 1$ ppm. This microwave cell presents a valve which allows the cavity to be filled with gas, which can be opened and close with a red knob. Then, the microwave cell filled with N_2 and with the valve closed was transferred outside the glovebox and connected to the vacuum line described above via such valve. In this way, the cavity could be filled with gas from the bottles and the perovskite thin film was exposed to it, while preventing the contact with ambient air at any time.

The procedure for the exposure to oxygen of the perovskite thin film was divided in the following steps:

1. At the initial step, valves **#1**, **#2**, **#3** and **#4** were closed. The vacuum line was usually left in high vacuum with the **vacuum valve** (**VV**) (turbo-pump mode) open to avoid any contamination.

2. After connecting the microwave cavity gas cell to the vacuum line, to ensure that there wasn't any leakage, the **VV** was closed and valve **#2** was opened to observe an increase of pressure in the line to ~ 45-50 mbar by the pressure indicator (PI). the **VV** (pre-pump mode) was opened to vacuum the system.
3. The **VV** was closed.
4. Subsequently, valve **#1** was also opened to observe an increase of pressure to ~ 55-70 mbar due to the gas contained in the microwave cell. The **VV** (pre-pump mode) was opened and used to vacuum the microwave cell and the whole system to ~ 10^{-1} mbar.
5. The **VV** and valve **#2** were closed.
6. Then, the process of flushing the line with the 21% O_2 / 79% N_2 gas mixture was started. The oxygen/nitrogen gas mixture bottle and valve **#3** were opened until reaching a pressure in the line of ~ 300-400 mbar. Then, valve **#3** was closed**.** Afterwards, the **VV** (pre-pump mode) was opened to remove such gas mixture. The **VV** was closed again. This cycle of flushing was repeated for three times before introducing the oxygen/nitrogen gas mixture in the line again with pressure of ~ 1 bar. Then, valve **#3** was closed, as well as the **VV** and the bottle of 21% O_2 / 79% N_2 gas mixture.
7. Valve **#2** was opened and the oxygen/nitrogen gas mixture filled the microwave cell (with a drop of pressure of ~ 10 %) containing the perovskite thin film, which was thus exposed to the 21% O_2 / 79% N_2 gas mixture left in the cell. The specific time of exposure to O_2 is monitored from this moment using a timer.
8. Then, valve **#1** and valve **#2** were closed.
9. The **VV** (first pre-pump mode, then turbo-pump mode) is opened and the line is vacuumed. The vacuum line is kept in high vacuum by leaving the **VV** (turbo-pump mode) opened.
10. The microwave cell filled with the 21% O_2 / 79% N_2 gas mixture is disconnected from the vacuum line and ready for any measurement after varying times of exposure to oxygen.
11. Afterwards such oxidation, the microwave cell is connected again to the vacuum line by following Step 1.
12. The **VV** (first pre-pump mode, then turbo-pump mode) was opened and used to vacuum the microwave cell and the whole system to 10^{-4} mbar in order to remove most of the O_2.
13. The **VV** and valve **#2** were closed.
14. Then, the process of flushing the line with N_2 gas was started. The N_2 gas bottle and valve **#4** were opened until reaching a pressure in the line of ~ 300-400 mbar. Then, valve **#4** was closed**.** Afterwards, the **VV** (pre-pump mode) was opened to remove such N_2 gas. The **VV** was closed again. This cycle of flushing was repeated for three times before introducing N_2 gas in the line again with pressure of 1 bar. Then, valve **#4** was closed, as well as the **VV** and the bottle of N_2 gas.
15. Valve **#2** was opened and N_2 gas filled the microwave cell containing the perovskite thin film, which was thus exposed to N_2 gas left in the cell. The specific time of rest in N_2 is monitored from this moment using a timer.
16. Repeat Step 6 and Step 7.
17. The microwave cell filled with N_2 gas is disconnected from the vacuum line and ready for any measurement after varying times of rest in N_2.

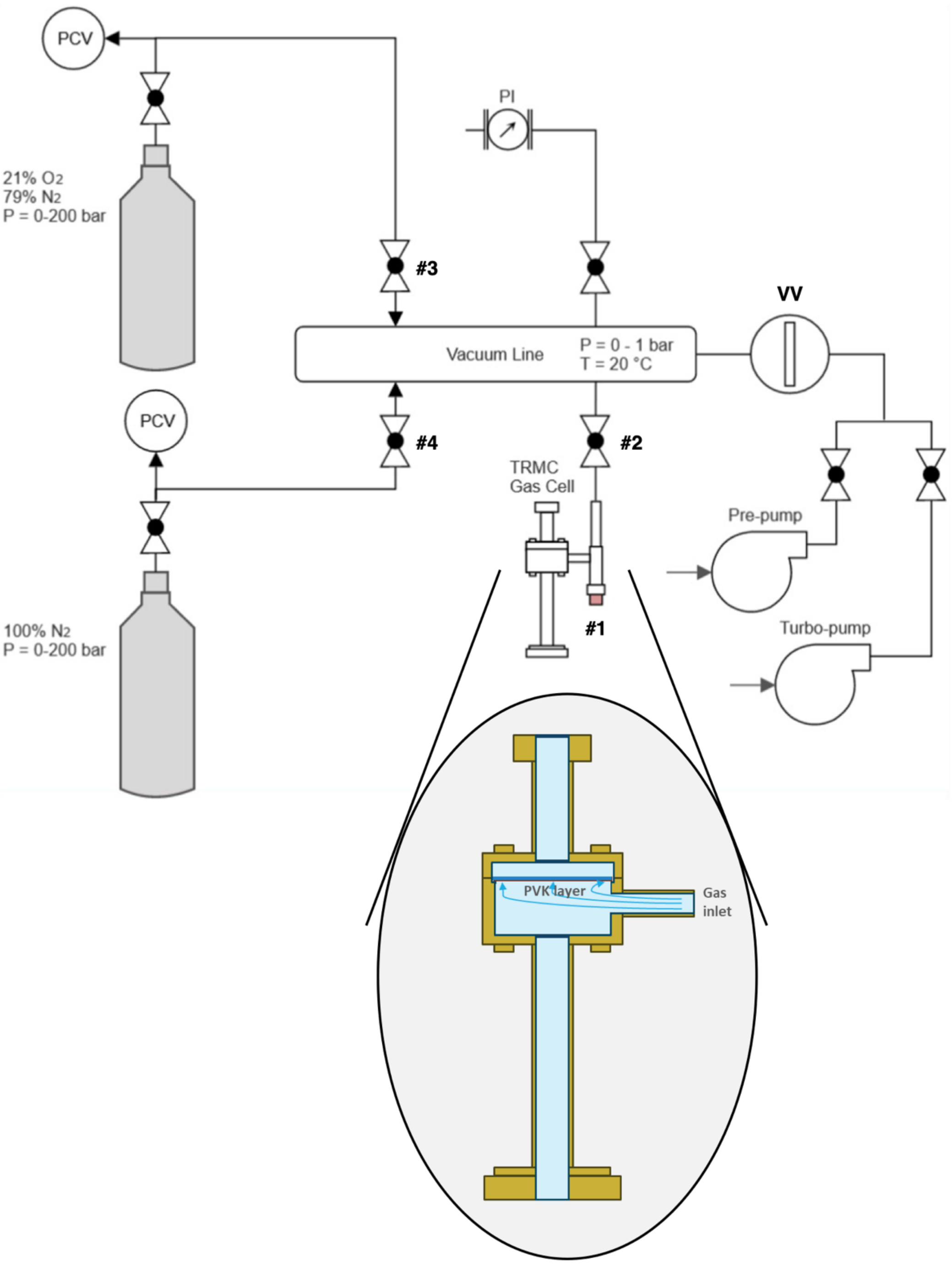

Figure S5: Schematic illustration of the vacuum line setup for oxidation of mixed Sn-Pb perovskite thin films. The vacuum line is connected to a specific microwave cavity gas cell, of which valve **#1** is part, containing the perovskite thin film under investigation. The microwave cell is connected to the line via valve **#2**. The **vacuum valve** (**VV**) connects to the line a vacuum pump for pre-pumping until a maximum of 10^{-2} mbar, a turbo-pump for high vacuum pumping until a maximum of 10^{-4}. A bottle of 21% O_2 / 79% N_2 gas mixture is connected via valve **#3** and a bottle of high-purity N6.0 N_2 gas is connected via valve **#4**. Both bottles present a main knob/handle to open them, pressure

and flow control valves and safety pressure control valves (PCV) to avoid overpressure in the system. It is possible to check the pressure in the line at any moment by the two pressure indicators (PI) connected to the vacuum line by a valve that stays always open.

For the experiments regarding the oxygen-induced doping, the SSMC measurements were carried out under the 21% O_2 / 79% N_2 gas mixture. For the experiments regarding the effect of resting in N_2, on the other hand, the SSMC measurements were performed under N_2 gas.

Moreover, we noticed that the laser light had an influence on the doping level of the perovskite thin film when the TRMC measurements were performed under the 21% O_2 / 79% N_2 gas mixture. This may be caused by the interaction between oxygen surrounding the perovskite thin film and light, which enhances the oxygen-induced doping. Therefore, all measurements with TRMC to study the oxygen-induced doping were performed in N_2, namely after filling the microwave cell with N_2 gas again, to avoid such effect, as it can be seen from **Figure S6.**

The perovskite thin film can also be removed at any moment from the microwave cavity gas cell inside a N_2-filled glovebox. Then, it can be studied with other characterization techniques.

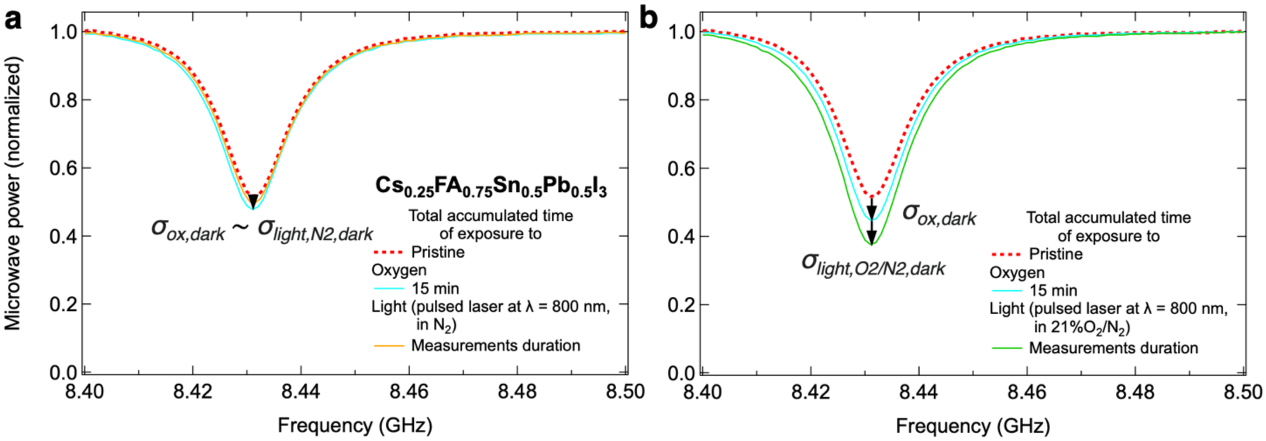

Figure S6: Effect of the laser light at λ = 800 nm used for TRMC measurements on the doping level of $Sn_{0.5}Pb_{0.5}$ perovskite thin film after causing oxygen-induced doping, depending on the gas left in the microwave cell. The laser intensity is in the order of ~ 1-100 nJ and the number of incident photons is in the order of ~ 10^9-10^{11} cm $^{-2}$ for each pulse. First, $\sigma_{0,dark}$ was measured in the dark for samples in pristine conditions (in red, dashed line). Next, the thin film was first exposed to the 21% O_2 / 79% N_2 gas mixture (in light blue, $\sigma_{ox,dark}$) and then measured by using the laser light of the TRMC under (a) N_2, after refilling the microwave cell with N_2 gas (in yellow), and (b) 21% O_2 / 79% N_2 gas mixture, by leaving it in the microwave cell after the oxygen-induced doping experiment (in green). No effect of the laser light is observed when the cell is filled again with N_2 gas ($\sigma_{ox,dark}$ ~ $\sigma_{light,N2,dark}$), except for a small reduction of σ_{dark} as an effect of resting in N_2. Conversely, when oxygen is left in the cell the laser light slightly increases the oxygen-induced doping level ($\sigma_{light,O2/N2,dark}$). We believe that the latter is caused by the interaction between oxygen

surrounding the perovskite thin film and light, which enhances the oxygen-induced doping. The oxygen is adsorbed or a bit absorbed by the film during the TRMC measurements and then it reacts with light.

E/M 6) Illumination setup

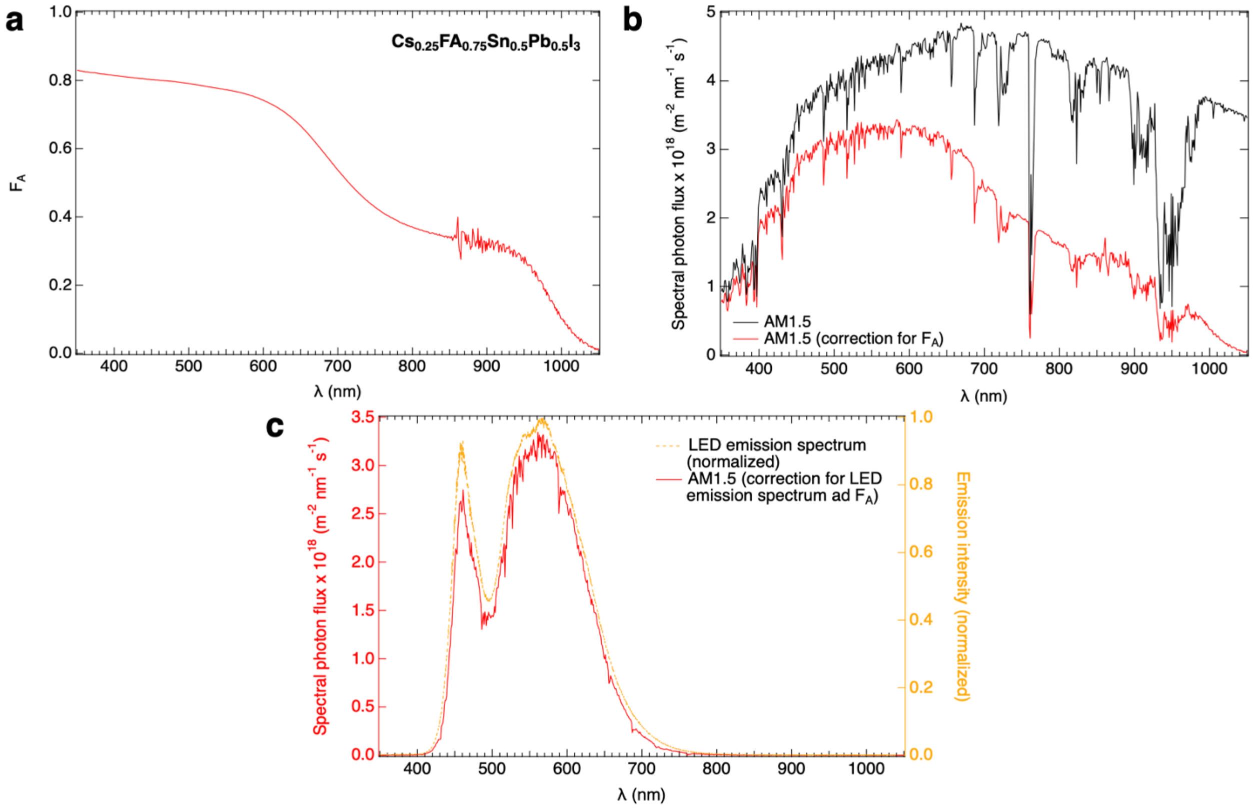

Figure S7: (a) Fraction of absorbed light (F_A) of a $Sn_{0.5}Pb_{0.5}$ perovskite thin film. (b) AM1.5 spectrum before (in black)[3] and after (in red) correction for F_A. (c) AM1.5 spectrum corrected for the emission spectrum of the white light LED lamp and F_A. The integration of such spectrum, along with knowing the power of the LED lamp in the visible light range in suns and the thickness of the layer, allows the number of photons absorbed by the perovskite thin film to be calculated.

The effect of the simultaneous exposure to oxygen and light was also investigated. In this case, the perovskite thin films were exposed to the 21% O_2 / 79% $N_{2\ 2}$ gas mixture to cause oxygen-induced doping while simultaneously being illuminated by a lamp. In this case, the 21% O_2 / 79% N_2 gas mixture is first introduced in the microwave cavity gas cell, after which the microwave cell is moved to the TRMC setup where it is illuminated by the lamp. A LuxSpot LS17-002D55 white light light-emitting diode (LED) lamp by LedEngin with emission spectrum in the range of visible light (λ = 400-700 nm) was used to illuminate the area of the perovskite thin films under the 21% O_2 / 79% N_2 gas mixture. A coherent LM-2 VIS silicon photodiode sensor was used to detect the incident light intensity. The perovskite

layers were irradiated with a power density of 51 ± 1 mW cm^{-2}, which corresponds approximately to ~ 1.2 suns in the visible range of light, at the surface of the sample for varying exposure times.

As shown in **Figure S7**, by correcting the intensity of the white light LED lamp by the wavelength-dependent fraction of absorbed light (F_A) of the perovskite thin films (taking a $Sn_{0.5}Pb_{0.5}$ perovskite layer as a reference) and integrating for the spectral emission wavelength range of the LED, i.e. λ = 400-700 nm, the number of absorbed photons was estimated to be equal to ~ 2.51 x 10^{21} cm^{-3} s^{-1} for a LED lamp power of ~ 25 ± 1 mW.

Moreover, the perovskite films were exposed for t ~ 24 h to only oxygen or simultaneously oxygen and light and subsequently stored in N_2 for *t* ~ 7 d (prolonged exposure treatments) to induce more significant changes in the perovskite and facilitate the detection of potential reaction products. In this specific case, the microwave cavity gas cell was left connected to the vacuum line filled with the 21% O_2 / 79% N_2 gas mixture while not being illuminated (long-term exposure to only oxygen) or being illuminated (long-term exposure to simultaneously oxygen and light) by the white light LED lamp as shown in **Figure S8**. Thus, despite the oxygen consumption by the oxidation reactions going on overnight, the oxygen supply is kept high by the extra volume of gas available inside the vacuum line, which is ten times larger than the volume of gas in the microwave cell.

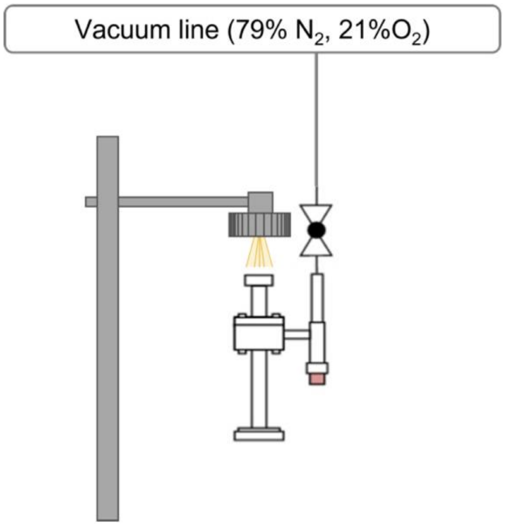

Figure S8: Schematic illustration of the setup used for prolonged exposure to simultaneously oxygen and light of perovskite thin films. For clarity, the original vacuum line scheme has been simplified to show only the relevant components of this specific setup.

E/M 7) Quasi-Fermi level splitting measurements and determination

The microwave conductivity setup was also used to determine the quasi-Fermi level splitting (QFLS) of the perovskite thin films under pristine conditions, after exposure to oxygen (or simultaneously oxygen and light) and after storage in N_2. The QFLS at AM 1.5 was determined by using a monochromatic green LED (λ = 522 nm) to create photo-

induced excess charge carriers. The LED light intensity corresponding to AM 1.5 was calculated by integrating the solar spectrum from higher photon energy down to 1.24 eV, corresponding to the bandgap of $Sn_{0.5}Pb_{0.5}$, resulting in a photon flux of ~ 2.3 x 10^{17} photons s^{-1} cm^{-2}. By using an optical sensor, the LED intensity was set to an irradiance of ~ 86 mW cm^{-2} to match such value. The LED light was modulated to a frequency of 1 Hz using a function generator. The change in voltage (ΔV) between dark (light off) and light (light on) over the microwave detector was probed by a lock-in amplifier. The QFLS can be calculated using **Equation (S8)**.[4]

$$QFLS = \frac{k_B T}{e} ln\left(\frac{(n_{e,dark} + \Delta n_e)(n_{h,dark} + \Delta n_h)}{n_i^2}\right) \quad (8)$$

Where the k_BT/e is the thermal voltage, n_i represents the intrinsic carrier density, $n_{e,dark}$ and $n_{h,dark}$ are respectively the dark electron and dark hole densities in thermal equilibrium, and Δn_e and Δn_h are respectively the photo-induced excess charge carrier densities.[4]

We calculated n_i = 5 x 10^7 cm^{-3} for mixed Sn-Pb perovskites by using **Equation (S9)**.[5]

$$n_i = \sqrt{N_C N_V} e^{(-\frac{E_g}{2k_BT})} \quad (9)$$

Where E_g is the perovskite band gap energy, k_B the Boltzmann constant, T is the temperature (298 K) and N_C and N_V are the effective density of states function in the conduction and valence band, respectively, calculated by using **Equations (S10)** and **(S11)**.[5]

$$N_C = 2\left(\frac{2\pi m_e^* k_B T}{h^2}\right)^{\frac{3}{2}} \quad (10)$$

$$N_V = 2\left(\frac{2\pi m_h^* k_B T}{h^2}\right)^{\frac{3}{2}} \quad (11)$$

Where h is the Planck constant and m_e^* = 0.16 and m_h^* = 0.15 are respectively the effective masses of electrons in the conduction band and holes in the valence band, reported in the literature for a perovskite with similar composition.[6]

From the SSMC measurements, we obtained σ_{dark} and, by applying Equation (2) in the main text, the corresponding value $n_{h,dark}$ for the perovskite thin films under different conditions, i.e. pristine, after exposure to oxygen (or simultaneously oxygen and light) and after storage in N_2. We calculated that $n_{h,dark}$ is in the order of ~ 10^{15}-10^{16} cm^{-3} for the layers under pristine conditions (and after restoring the initial $\sigma_{0,dark}$ after storage in N_2), while it is in the order of ~ 10^{16}-10^{17} cm^{-3} after exposure for oxygen for t ~ 1 h 30 min and exposure to simultaneously oxygen and light for t ~ 15 min. These values are in the order of magnitude or higher than that of Δn_e and Δn_h under AM 1.5, which is in the order of 10^{16} cm^{-3} at the intensity of 1 sun. On the other hand, the value for $n_{e,dark}$ is orders of magnitude smaller

than Δn_e. Besides, since the absorption of each photon leads to the generation of one free electron and one free hole, we considered $\Delta n_e = \Delta n_h = \Delta n$. Therefore, **Equation (S8)** can be simplified in **Equation (S12)**.

$$QFLS = \frac{k_B T}{e} ln\left(\frac{\Delta n \cdot n_{h,dark} + \Delta n^2}{n_i^2}\right) \tag{12}$$

The change in voltage ΔV caused by the LED light was measured by the lock-in amplifier, together with the cell voltage (V) and the n factor connecting the change in voltage and the change in microwave power. The change in microwave power was calculated by **Equation (S13)**.[6]

$$n \cdot \frac{\Delta V}{V} = \frac{\Delta P}{P} \tag{13}$$

As shown before, the change in microwave power is used to calculate the change in conductivity by using **Equation (S1)**. Then, the change in conductivity is used to calculated Δn by using **Equation (S4)**, with the difference that we do not calculate $\Delta n_{h,dark}$ using μ_h, but the total LED light-induced carriers Δn using μ).

E/M 8) Other characterization techniques

The perovskite thin films were measured under pristine conditions, after exposure to oxygen, after simultaneous exposure to oxygen and light and after resting in a N_2 for a period of time. Considering the metastable nature of oxygen-induced (light-enhanced) doping, elemental and optoelectronic analyses were performed immediately after exposure to oxygen (or oxygen and light). The total accumulated time of exposure to oxygen (or oxygen and light) and rest under N_2 at the moment of the measurement was always specified. When possible, the material characterizations were carried out in vacuum or in air-tight holders filled with N_2. When the material characterizations were carried out in ambient air, some samples were only produced for the purpose of these measurements or, when not possible, these were the last measurements conducted for any given sample. In any case, each perovskite thin film was brought from the glovebox to the specific characterization instrument within an air-tight holder or in the shortest possible time (< 2 min) to minimize the exposure to ambient air during the acquisition of information and reduce the influence on the results.

X-ray diffraction The X-ray diffraction (XRD) analysis of the samples under pristine conditions was carried out by using a Bruker D8 Advance-ECO X-ray diffractometer, equipped with a Cu-K_α X-ray source (λ = 1.542 Å) operating at 40 kV and 25 mA and a Lynxeye-XE-T 1D position-sensitive energy-discriminative detector. The measurements have been carried out in Bragg-Brentano geometry with a fixed sample illumination of 3.0 mm for a range of angles 2θ = 5°-60°, step size of 0.01° and a measuring time of 0.01 s/step. For the analysis of the changes in the X-ray diffraction patterns before and after prolonged exposure treatments, a Bruker D8 Discover X-ray diffractometer was used. This diffractometer is equipped with Twin/Twin optics, with a Cu-K_α (λ = 1.542 Å) operating at 40 kV and 40 mA. For these measurements, a Lynxeye-XE position-sensitive detector operated in 1D high resolution mode, a Ni

foil filter to reduce the Cu-K_β radiation from the source were used with a fixed sample illumination of 6.0 mm for a range of angles $2\theta = 5°$-$60°$, step size of 0.02° and a measuring time of 1.2 s/step. The sample was located inside an Anton Paar PAAR XRK900 reactor chamber for non-ambient conditions. This chamber is equipped with Be windows and the vacuum of $P < 10^{-4}$ mbar was continuously monitored and the temperature of $T = 25 \pm 1$ ºC controlled with an Anton Paar TCU250 control unit. The same instrument and reactor chamber have been used for Grazing Angle X-ray Diffraction (GIXRD) measurements. In this case, on the primary side a Goebel Mirror was used to create a parallel X-ray beam and a fixed 0.1 mm slit to control the footprint on the sample. On the secondary side, a 0.2° Soller slit was used in combination with the Lynxeye-XE detector operating in 0D high-count rate mode. The measurements have been carried out with an incident angle $\omega = 0.5º$, while $2\theta = 5°$-$60°$ was achieved by varying the detector angle in 0.02° steps with 10s/step. Before the measurements commenced, the height and angle of the sample were carefully aligned.

UV-Vis-NIR spectroscopy The optical properties (absorption, transmission and reflection) of the samples were measured by a PerkinElmer LAMBDA 1050+ UV/Vis/NIR spectrophotometer with a 150 mm integrating sphere. All the samples were transferred to the UV-Vis-NIR spectroscopy setup by means of an air-tight sample holder and immediately measured after being removed from the holder and placed in the setup, to minimize the effect of the exposure to ambient air. Due to such exposure, this was the last characterization measurement conducted for a given sample.

Profilometry The average thickness of the thin films was determined by measurements performed with a Veeco/Bruker Dektak 8 Stylus Profilometer with a stylus tip diameter of 12.5 μm and a force (load) of 5 mg (≃ 50 μN). This is a destructive measurement performed in ambient air, so it was the last characterization measurement conducted for a given sample.

X-ray photoelectron spectroscopy The elemental composition and chemical state analyses were carried out by X-ray photoelectron spectroscopy (XPS) measurements. These were performed by using a Thermo Scientific K-Alpha system, incorporating an X-ray gun based on an Al K_α radiation source with energy of 1486 eV and a spot size kept at the default value of 800x400 μm^2. The samples were transferred in the XPS setup by means of an air-tight sample holder and a load lock preventing the contact with ambient air. All measurements were conducted in ultra-high vacuum conditions ($P < 2\ 10^{-9}$ mbar). A flood gun operating at 0.15 mA and 1 V was used to replenish the electrons emitted from the sample surface from the system to hinder charging during the measurement. Depth profiling was conducted by analyzing the elemental composition of the thin film while etching it with an argon-based ion beam. The films ware etched with an ion beam with energy $E = 1$ keV. In spite of selecting a low ion beam energy, it is important to mention that unavoidable charging of the pristine perovskite films occurred during the measurements, likely due to the impossibility to compensate with the flood gun the favorable oxidation of Sn^{2+} to Sn^{4+} and the loss of electrons from the perovskite layer. Consequently, the XPS peaks shifted in binding energy, broadened and deformed, especially during etching, for all elements analyzed. The corresponding XPS atomic % and depth profiling is a careful

extrapolation from those data. For such pristine samples suffering from charging, it was still possible to fit the peaks and generate a depth profile of the different elements for the first 12 steps of etching. For all films, peak fitting of the detailed $Sn3d_{5/2}$ and O1s spectra was carried out only before etching (t_{etch} = 0 s), namely at the film surface to limit the influence of the aforementioned charging which is stronger during etching, in order to observe the relative oxidation species.

Steady State Microwave Conductance (SSMC) – Dark conductivity & Time-resolved Microwave Conductivity (TRMC) – Photogenerated charge carrier dynamics

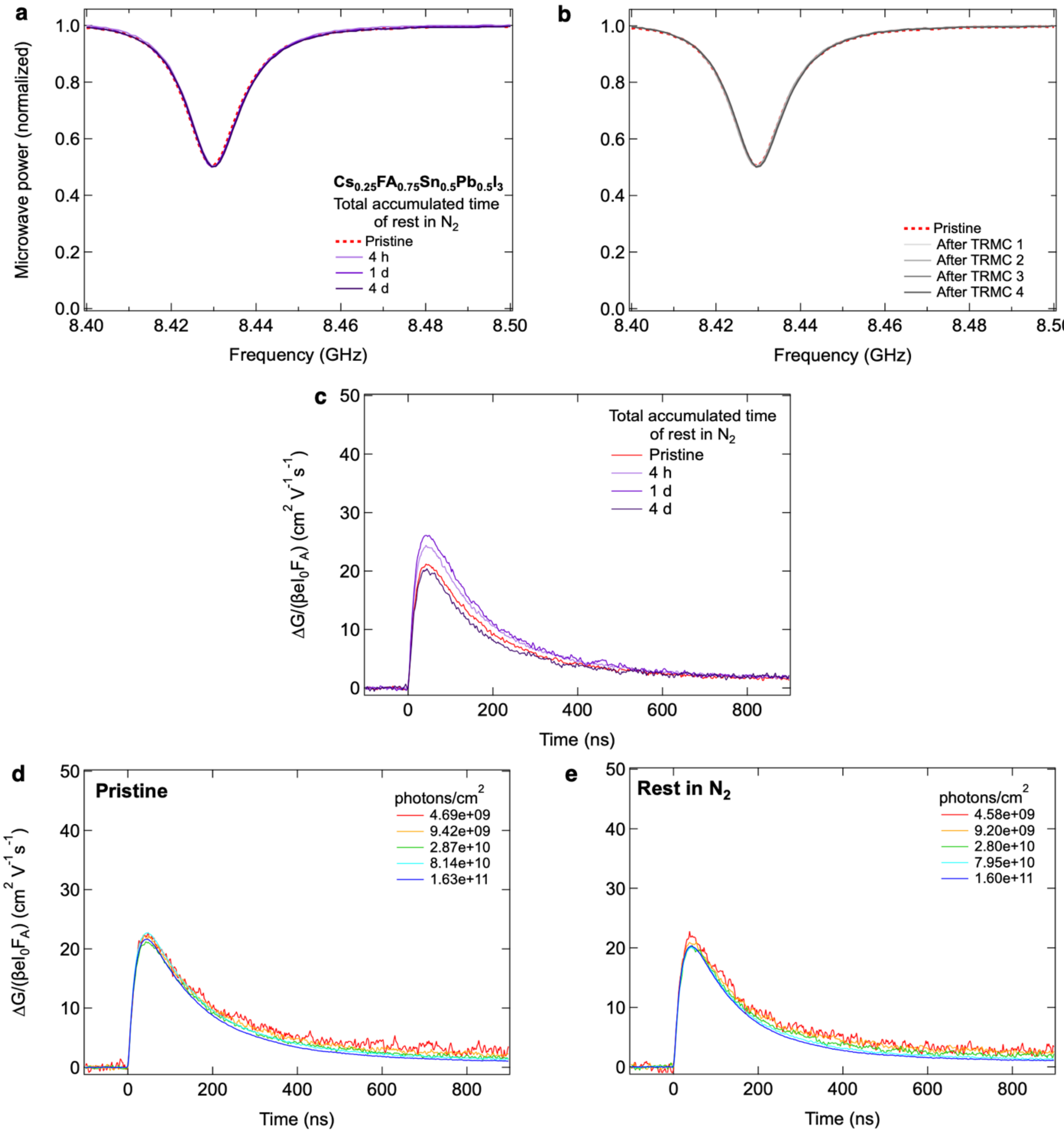

Figure S9: Comparison of SSMC measurements and TRMC measurements showing the effect of resting in N_2 on a $Sn_{0.5}Pb_{0.5}$ thin film. (a) Background conductivity measured in the dark for a film in pristine conditions (in red) and after resting in N_2 for varying time intervals (in purple) and (b) effect on the dark conductivity of a film left to rest in N_2 for varying time intervals of the laser light at λ = 800 nm used for several TRMC measurements. It is worth noting that resting in N_2 does not induce any change in $\sigma_{0,dark}$. Moreover, also the laser light does not induce any change

$\sigma_{0,dark}$, even after multiple TRMC measurements carried out over days. (c) Charge carrier dynamics for a film in pristine conditions (in red) and after resting in N_2 for varying time intervals (in purple). The TRMC traces in (c) were recorded at short time scale (100 ns) using identical laser intensities (~ 2-3 x 10^{10} photons cm^{-2}) and wavelength (λ = 800 nm). It can be seen that resting in N_2 does not induce any change in the charge carrier dynamics, except for small variations in the maximum TRMC signal deriving from laser power fluctuations. (c) TRMC measurements for a perovskite film in (d) pristine conditions and (e) after resting in N_2 for t = 4 d. The TRMC traces in (d,e) were measured at short time scale (100 ns), with different laser intensities and at the same wavelength (λ = 800 nm). As mentioned before, resting in N_2 does not induce any change in the charge carrier dynamics. All the TRMC traces were corrected for the correct K factor calculated depending on the corresponding level of background conductivity. Note that the TRMC traces are also corrected for the absorbed fraction of light in **Figure S21** at the excitation wavelength λ = 800 nm to derive the product between the charge carrier yield and mobilities sum.

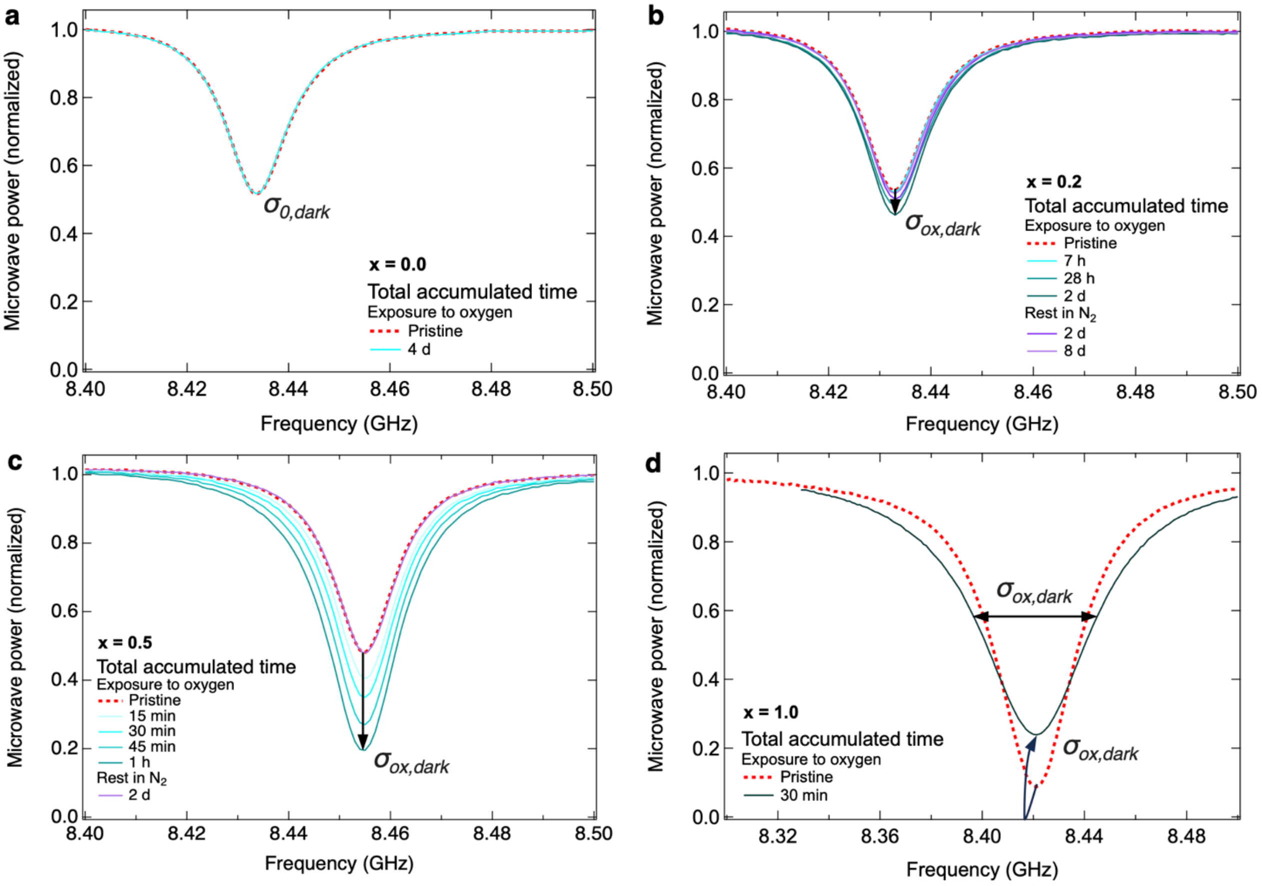

Figure S10: SSMC measurements showing the metastable oxygen-induced doping of $Cs_{0.25}FA_{0.75}Sn_xPb_{1-x}I_3$ with tin fractions (a) x = 0.0, (b) x = 0.2, (c) x = 0.5 and (d) x = 1.0. The background conductivity was measured in the dark for samples in pristine conditions (in red, dashed line), during exposure to oxygen for varying times (in light blue) and after rest time under N_2 (in purple). Exposure to oxygen leads to different oxygen-induced doping rates in the samples, implying different susceptibilities to the phenomenon depending on the tin fraction. For Sn_0Pb_1, no increase in $\sigma_{0,dark}$ is observed even after t = 4 d of exposure to oxygen. For $Sn_{0.2}Pb_{0.8}$ and $Sn_{0.5}Pb_{0.5}$, an increase to $\sigma_{ox,dark}$ is observed

after exposure to oxygen for varying time intervals. Interestingly, oxygen-induced doping occurs at a faster rate for $Sn_{0.5}Pb_{0.5}$ than for $Sn_{0.2}Pb_{0.8}$. This is also the case for the restoration to $\sigma_{0,dark}$ when the film is left to rest in N_2, which takes place more rapidly for $Sn_{0.5}Pb_{0.5}$ than for $Sn_{0.2}Pb_{0.8}$. For the $Cs_{0.25}FA_{0.75}SnI_3$ perovskite, it must be point out that the initial $\sigma_{0,dark}$ is already significant even before any exposure to oxygen, which means that the pristine layers with tin fraction Sn_1Pb_0 are already substantially doped. We ascribed it to the properties of pure Sn-based perovskite, which make tin oxidation from Sn^{2+} to Sn^{4+} and self-doping even more likely to occur.[4] The initially high level of doping in such perovskite thin films may be due to the presence of some Sn^{4+} impurities in the SnI_2 precursor,[5,6] the easy oxidation of Sn^{2+} to Sn^{4+} during the perovskite precursors solution preparation due to the small concentration of oxygen present in the N_2-filled glovebox,[5,7] and the favorable formation of tin vacancies in pure Sn-based perovskite, which makes tin oxidation even more favorable.[4,8] It is clear that the addition of only SnF_2 is not enough to counteract such effects. After exposure to oxygen for t = 30 min, $\sigma_{ox,dark}$ reaches such a high level that the microwave electric field is reflected back by the film. Because of the high concentration of background free holes, the correspondent frequency dip gets broader and less deep due to the strong microwave reflection. This complicates the analysis of pure Sn-based perovskite. Hence, thorough investigation of the oxygen-induced doping in pure Sn-based perovskite was not practicable because the significantly high initial background conductivity easily led to falling outside the sensitivity window of SSMC measurements when the sample was further doped by exposure to oxygen.

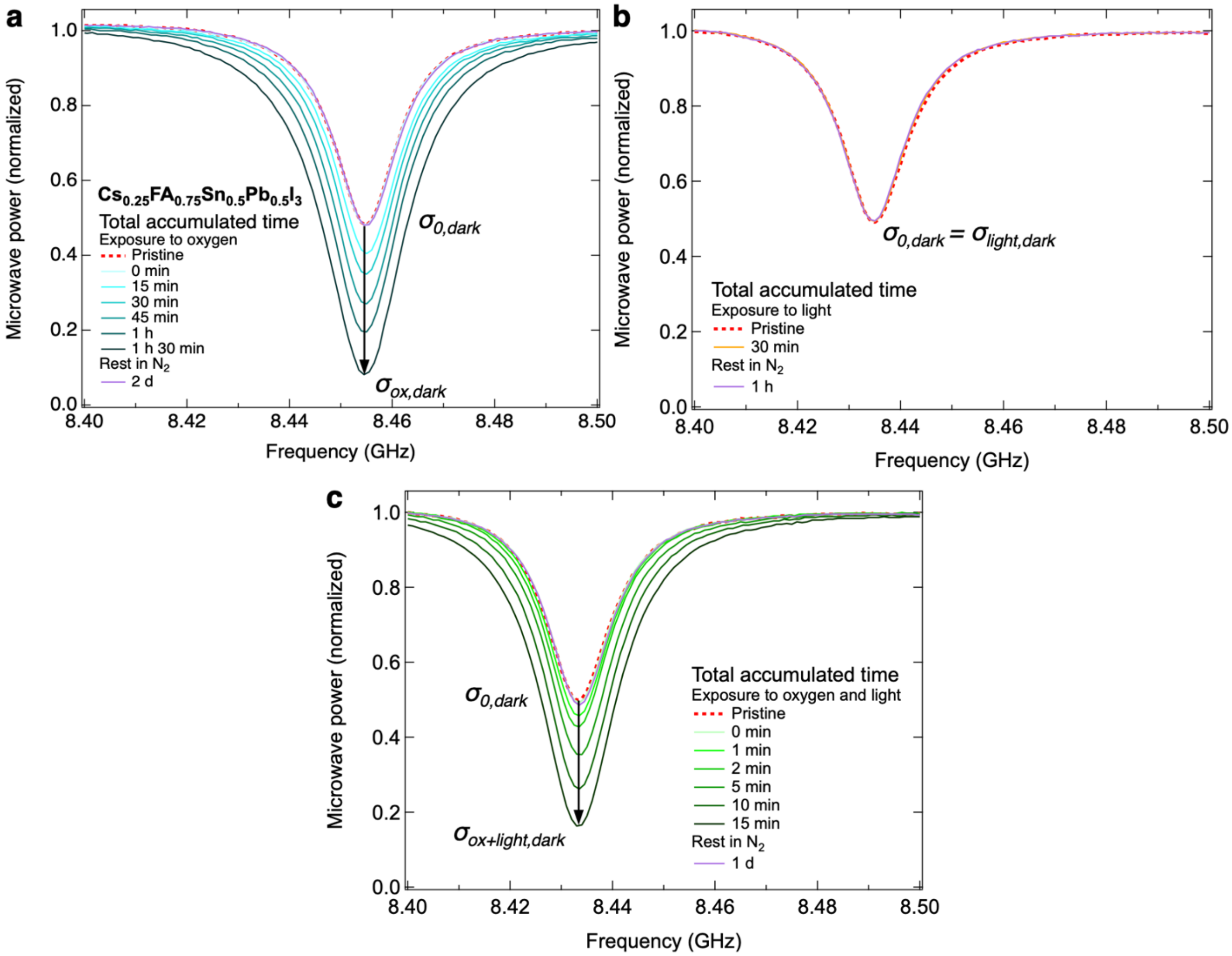

Figure S11: SSMC measurements showing the doping effect of exposure to oxygen, light or simultaneously oxygen on $Sn_{0.5}Pb_{0.5}$ perovskite thin films. The background conductivity was measured in the dark for samples in pristine conditions (in red, dashed line) and (a) during exposure to oxygen for varying times (in light blue, $\sigma_{ox,\mathrm{dark}}$), (b) after exposure to light (in yellow, $\sigma_{light,\mathrm{dark}}$), (c) after simultaneous exposure to oxygen and light (in green, $\sigma_{ox+light,\mathrm{dark}}$) and (d) after resting under N_2 (in purple, $\sigma_{0,\mathrm{dark}}$). Exposure to oxygen leads to an increase of the background conductivity to $\sigma_{ox,dark}$, a phenomenon defined as oxygen-induced doping, while exposure to light alone does not lead to doping ($\sigma_{0,\mathrm{dark}} = \sigma_{light,\mathrm{dark}}$). Surprisingly, the simultaneous exposure to oxygen and light strongly increases the rate of doping, reaching a level $\sigma_{ox+light,dark}$. Hence, it can be inferred that light enhances oxygen-induced doping.

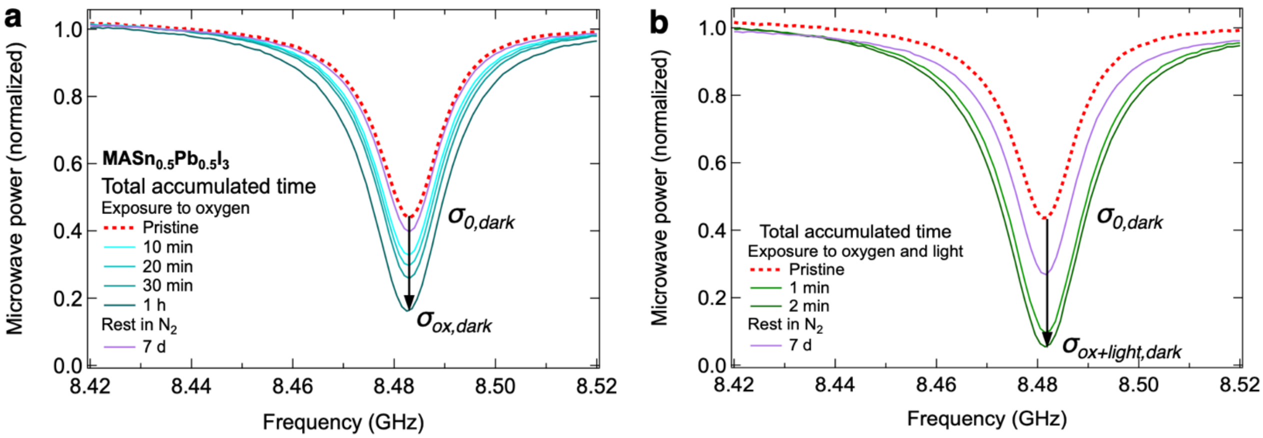

Figure S12: SSMC measurements showing the metastable (a) oxygen-induced doping and (b) light-enhanced oxygen-induced doping of $MASn_{0.5}Pb_{0.5}I_3$ showing the effect of changing the cation in the A sites on σ_{dark}. The background conductivity was measured in the dark for samples in pristine conditions (in red, dashed line), during exposure to oxygen for varying times (in light blue), after simultaneous exposure to oxygen and light (in green) and after rest time under N_2 (in purple). It can be noticed that the MA-based perovskite thin film presents higher oxygen-induced doping rates. The same applies when the film is exposed simultaneously to oxygen and light. On the other side, the restoring of $\sigma_{0,dark}$ occurs on longer time scales in both cases, since the background conductivity level is not back to $\sigma_{0,dark}$ even after 7 days of rest in N_2. Hence, it should be noted that the occurrence of the oxygen-induced doping phenomenon is independent on the choice of the A-site cation, with or without simultaneous illumination, although we acknowledge that its kinetic is different, as well its potential reaction products.

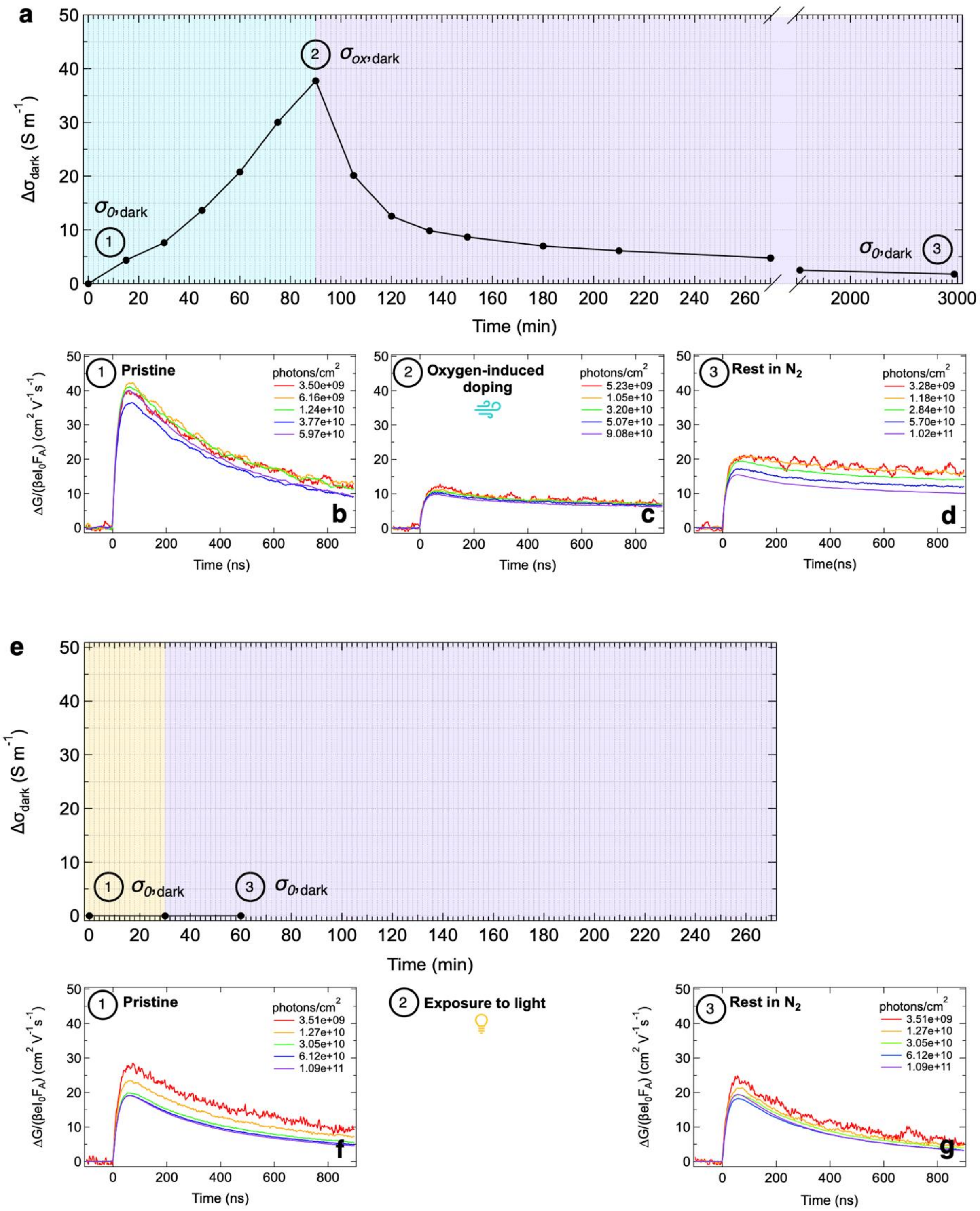
a
Δσ_dark (S m^-1)
Time (min)
σ_0,dark
σ_ox,dark
Pristine
Oxygen-induced doping
Rest in N2
photons/cm^2
ΔG/(βeI0FA) (cm^2 V^-1 s^-1)
Time (ns)
b
c
d
e
Exposure to light
f
g

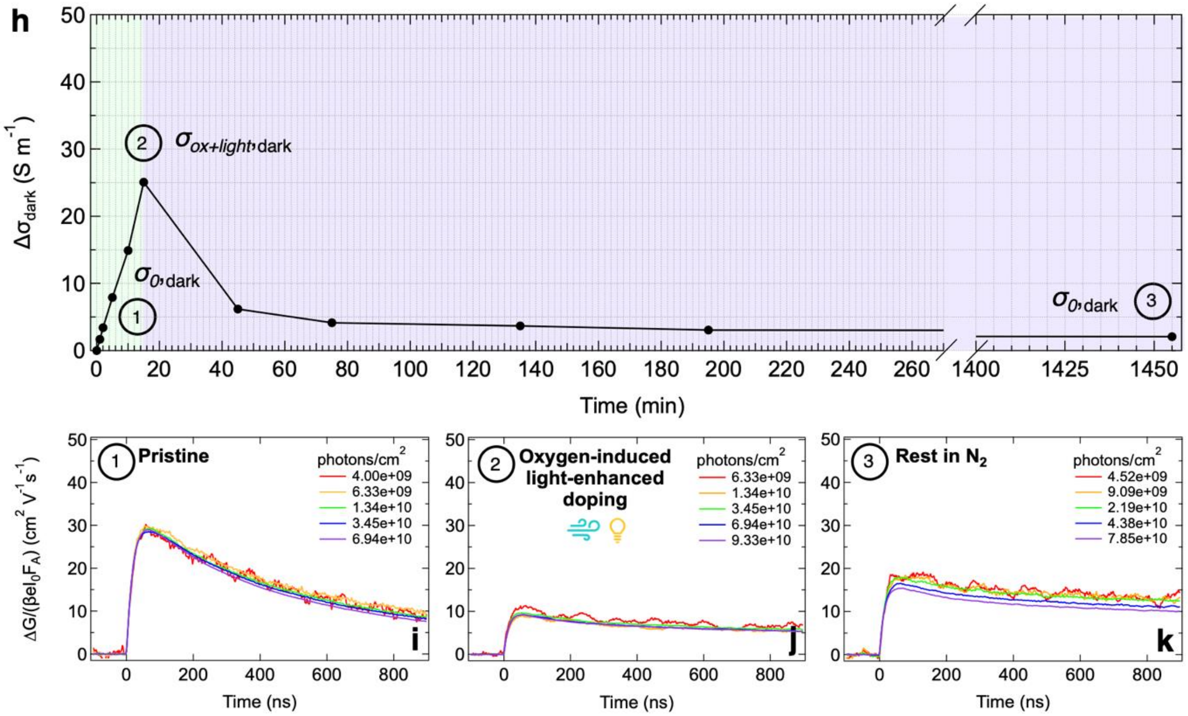

Figure S13: Evolution over time of σ_{dark} and charge carrier dynamics at different levels of σ_{dark} for $Sn_{0.5}Pb_{0.5}$ perovskite thin films. Evolution over time of doping showing the initial background conductivity $\sigma_{0,dark}$, its variation induced by exposure to (3a) oxygen $\sigma_{ox,dark}$, (3e) light or (3h) simultaneously oxygen and light $\sigma_{ox+light,dark}$, and its reduction back to $\sigma_{0,dark}$ after resting under N_2. For each sample the TRMC traces at short time scale (100 ns) were recorded using varying laser intensities at the same wavelength (λ = 800 nm). The TRMC traces are provided (3b, 3f, 3i) under pristine conditions, (3c, 3j) after exposure to oxygen for t = 1h 30 min or after simultaneous exposure to oxygen and light for t = 15 min and (3d, 3g, 3k) after resting under N_2 until the initial level $\sigma_{0,dark}$ was restored. Regarding the exposure to only light, only the TRMC trace after resting in N_2 for a short time has been recorded to avoid measuring the contribution of the photoconductance, showing however that the exposure to light for t = 30 min didn't produce any permanent change in σ_{dark}. The TRMC traces were corrected for the correct K factor calculated depending on the corresponding level of background conductivity. Note that the TRMC traces are also corrected for the absorbed fraction of light in **Figure S21** at the excitation wavelength λ = 800 nm to derive the product between the charge carrier yield and mobilities sum.

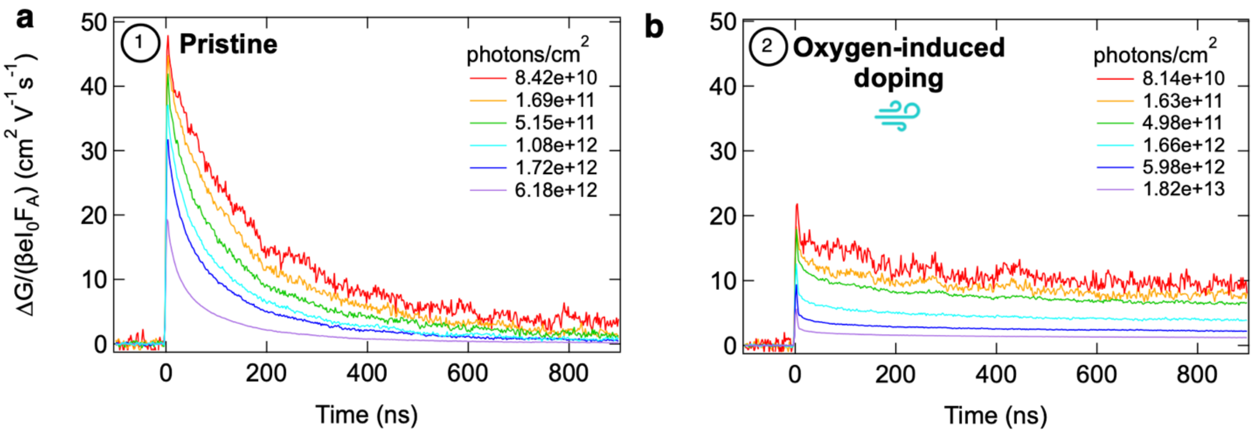

Figure S14: TRMC measurements performed with a microwave open cell for a $Sn_{0.5}Pb_{0.5}$ perovskite thin film, showing the charge carrier dynamics at short time scale (100 ns) (a) under pristine conditions and (b) after exposure to oxygen for a time interval of t = 1 h 30 min. The microwave cavity cell and open cell have an instrumental response time of respectively 18 ns and 2 ns. The latter allows to observe the early charge carrier dynamics before the charge carrier recombination occurs, which explains the higher TRMC signal for the open cell TRMC measurements. However, its lower sensitivity requires to use higher laser intensities. The use of high photon fluences also affect the intensity-dependent behaviour of the TRMC traces. In fact, when the dynamics is dominated by second-order recombination, the TRMC signal becomes progressively lower and its decay faster with increasing laser intensity, due to the corresponding increasing concentration of photogenerated carriers annihilating each other via band-to-band recombination. Hence, the open cell TRMC traces measured at high photon fluences show a stronger second-order recombination behavior compared to those measured by microwave cavity cell in **Figure S13**. The open cell TRMC traces were corrected for K = 1,000. Note that the TRMC traces are also corrected for the absorbed fraction of light in **Figure S21** at the excitation wavelength λ = 800 nm to derive the product between the charge carrier yield and mobilities sum.

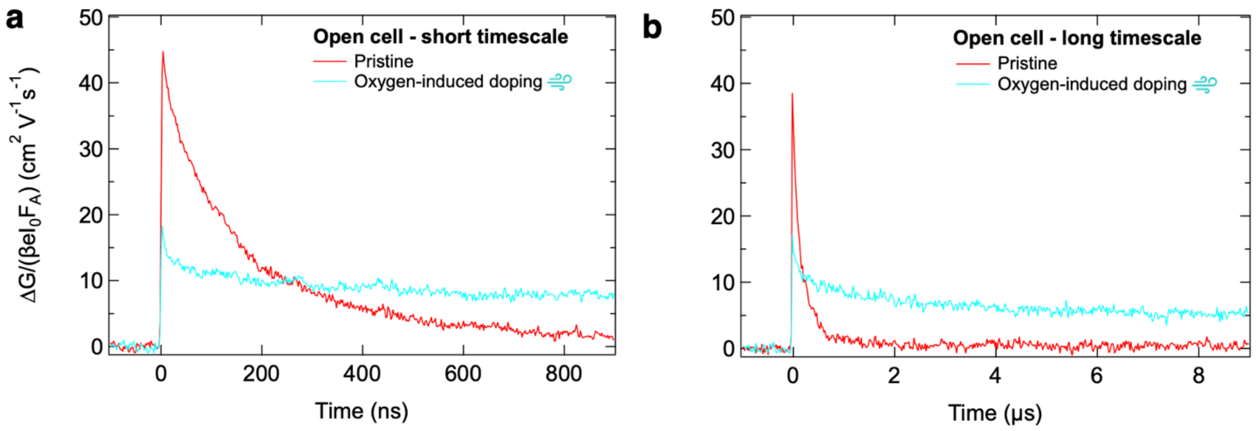

Figure S15: Comparison of charge carrier dynamics at (a) short time scale (100 ns) and (b) long time scale (1 μs) of $Sn_{0.5}Pb_{0.5}$ perovskite thin films measured under pristine conditions (in red) and after exposure to oxygen (in light blue) for a time interval of t = 1 h 30 min by using a microwave open cell. For each sample the TRMC traces were recorded using identical laser intensities (~ 1-2 x 10^{11} photons cm^{-2}) and wavelength (λ = 800 nm). The TRMC traces measured by microwave open cell were corrected for K = 1,000. Note that the TRMC traces are also corrected for the absorbed fraction of light in **Figure S21** at the excitation wavelength λ = 800 nm to derive the product between the charge carrier yield and mobilities sum.

Quantification of tin oxidation

Considering $\Delta\sigma_{dark}$ corresponding to a moderate doping level of $\Delta n_{h,dark}$ ~ 6.3 x 10^{16} cm^{-3} and knowing from Equation (3) in the main text that each oxygen molecule reacting with tin leads to the creation of two free holes, the concentration of O_2 reacting with an equivalent amount of Sn^{2+} is calculated in **Equation (S14)**.

$$n_{O_2} = \frac{\Delta n_{h,dark}}{2} \sim 3.1\ x\ 10^{16}\ \ cm^{-3} \tag{14}$$

The density of tin in the perovskite crystal structure, assuming for simplicity all Sn^{2+} in the perovskite crystal structure, is shown in **Equation (S15)**.

$$\rho_{Sn^{2+}} = \frac{m_{Sn^{2+}}}{V_{cell}} = \frac{\frac{0.5 \cdot P_{A,Sn^{2+}}}{N_A}}{a^3} = 0.399\ g\ cm^{-3} \tag{15}$$

Where the atomic weight of tin is $P_{A,Sn2+}$ = 118.71 g mol^{-1}, N_A is the Avogadro's number (N_A = 6.022 x 10^{23} mol^{-1}) and V_{cell} is the volume of the unit cell, calculated assuming a pseudo-cubic perovskite crystal structure with lattice parameter a obtained by applying **Equation (S16)** after performing the XRD diffraction analysis on the $Sn_{0.5}Pb_{0.5}$ perovskite film, shown in **Figure S16**:[7]

$$a = \frac{\lambda}{2 \cdot sin\theta}\sqrt{(h^2 + k^2 + l^2)} = 6.276\ \text{Å} \tag{16}$$

Where $sin\theta$ is the sine of half the diffraction angle 2θ at which a perovskite XRD peak with Miller indeces (hkl) is located, in this case the (001) reflection at $2\theta \simeq 14.1°$, and λ = 1.54056 Å is the wavelength of the Cu-K_α X-rays used in these measurements.

It follows that the molar density of Sn^{2+} in the perovskite crystal structure can be obtained, as shown in **Equation (S17)**.

$$n_{Sn^{2+}} = \frac{\rho_{Sn^{2+}} \cdot N_A}{P_{A,Sn^{2+}}} \sim 2.02\ x\ 10^{21}\ \ cm^{-3} \tag{17}$$

Hence, when comparing n_{O2} and n_{Sn2+}, it is possible to notice that only a few millionths of tin need to be oxidized and form tin oxide species SnO_x, such as SnO_2, to reach such doping level. This corresponds to the change of a minuscule fraction in the crystal structure.

X-Ray Diffraction (XRD) – Crystal structure

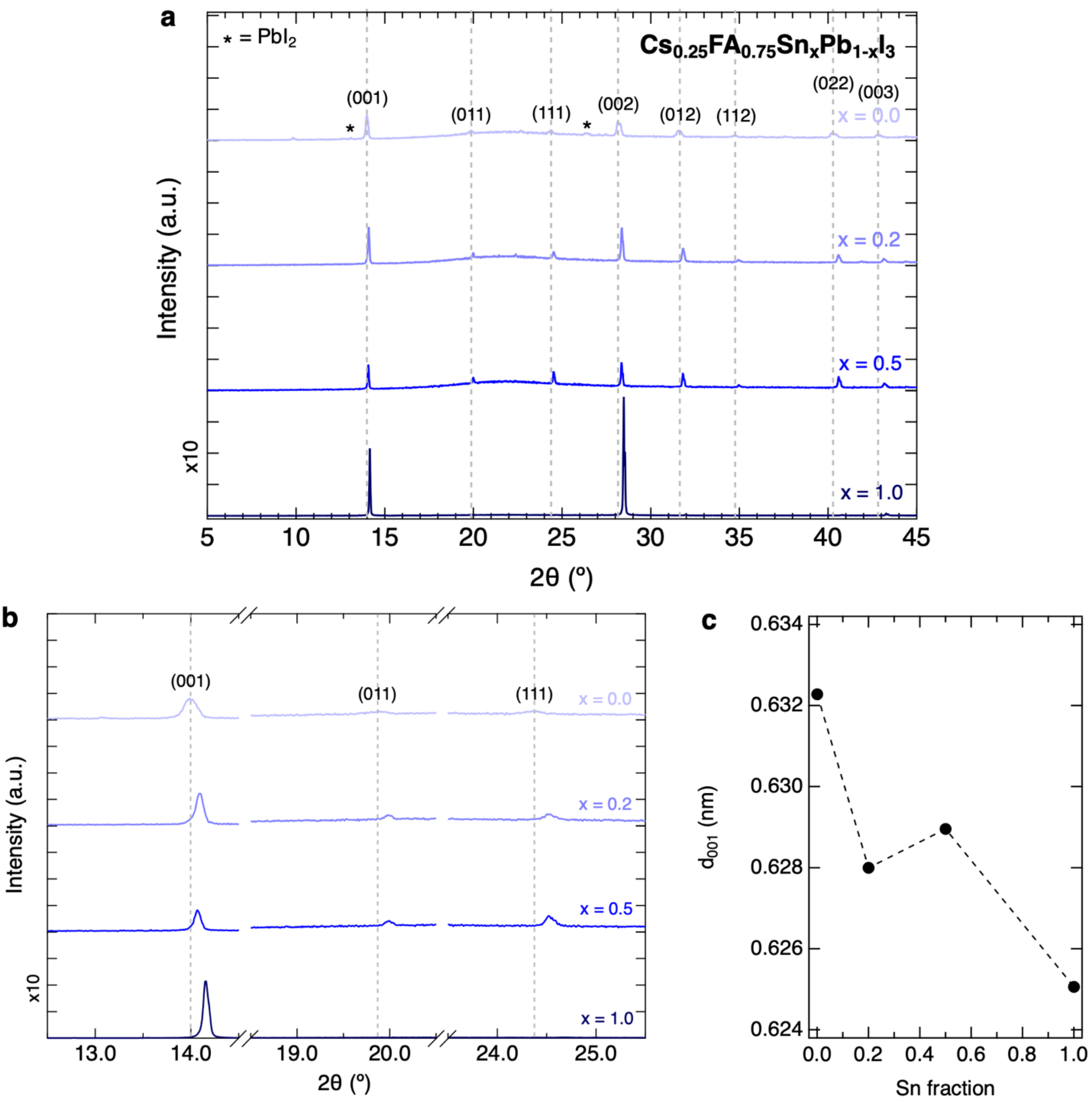

Figure S16: Crystal structure properties of $Cs_{0.25}FA_{0.75}Sn_xPb_{1-x}I_3$ perovskite thin films with tin fractions *x* = 0.0, *x* = 0.2, *x* = 0.5 and *x* = 1.0. (a) XRD diffraction patterns showing the Miller indices of the characteristic diffraction peaks of the pseudocubic crystal phase of perovskite, (b) position of the three perovskite peaks corresponding to different sets of crystal planes (001) at $2\theta \simeq 14.0°$, (011) at $2\theta \simeq 19.9°$, and (111) $2\theta \simeq 24.4°$,[8,9] and c) interplanar distance between the (001) planes, d_{001}, as a function of the Sn fraction, calculated by using the position of the (001) peak and the Bragg's law.[10] It is possible to notice an excess of PbI_2 for the Sn_0Pb_1 film, as shown for similar compositions in literature.[8,11,12] The bump between $2\theta \simeq$ 20-25° derives from the quartz substrate on which the perovskite film is

deposited. Moreover, the diffraction intensity of the perovskite peaks remains relatively unaltered in all compositions, with the exception of the Sn_1Pb_0 film, whose peaks present a considerably higher intensity despite using the same measurements parameters (note the x10 rescaling on the corresponding y-axis). For Sn_0Pb_1, $Sn_{0.2}Pb_{0.8}$, $Sn_{0.5}Pb_{0.5}$, it can be pointed out that the overall similar intensity of the different perovskite diffraction peaks appearing in each diffraction pattern indicates isotropic grain growth, showing hardly any evidence of preferential orientation.[8,11,12] On the other hand, the film Sn_1Pb_0 containing only Sn shows a preferential grain orientation, given that its diffraction pattern has only two high-intensity perovskite peaks and a less intense peak corresponding respectively to the sets of planes (001), (002) and (003), which are all parallel to each other. No other perovskite peaks corresponding to other sets of planes are present in the diffraction pattern of the Sn_1Pb_0 film. Furthermore, it is worth mentioning that the characteristic perovskite peaks progressively shift to higher diffraction angles with increasing tin content, in spite of the $Sn_{0.5}Pb_{0.5}$ film which shows a shift to lower diffraction angles. The ionic radius of Sn^{2+} ($r_{Sn,eff}$ = 110 pm) is smaller than the one of Pb^{2+} ($r_{Pb,eff}$ = 119 pm),[13] which in turn leads to a smaller interplanar distance between the atomic planes and an overall contraction of the crystal lattice. In this case, the perovskite diffraction peaks should shift to the right for compositions with increasing tin content according to Bragg's Law,[10] as reported in literature for perovskites made of similar constituents.[8,9,11] Since d_{001} does not decrease linearly with higher Sn fraction, it is possible to state that the contraction of the perovskite crystal lattice as a function of Sn fraction does not follow the empirical Vegard's law, which assume a linear relationship between the lattice constants of an alloy and its composition.[14] The deviation from the Vegard's law observed for the $Sn_{0.5}Pb_{0.5}$ film is most likely a consequence of compositional heterogeneity due to the faster crystallization dynamics in solution of Sn-based perovskite with respect to its Pb counterpart.[15-17] This may lead to macrostrains on the crystal lattice due to an enrichment of Sn at the surface of the layer during the spin coating synthesis,[17] as observed also in the XPS depth profiling in **Figures S23f** and **S23g**, which affect the position of the perovskite peaks in the diffraction patterns.

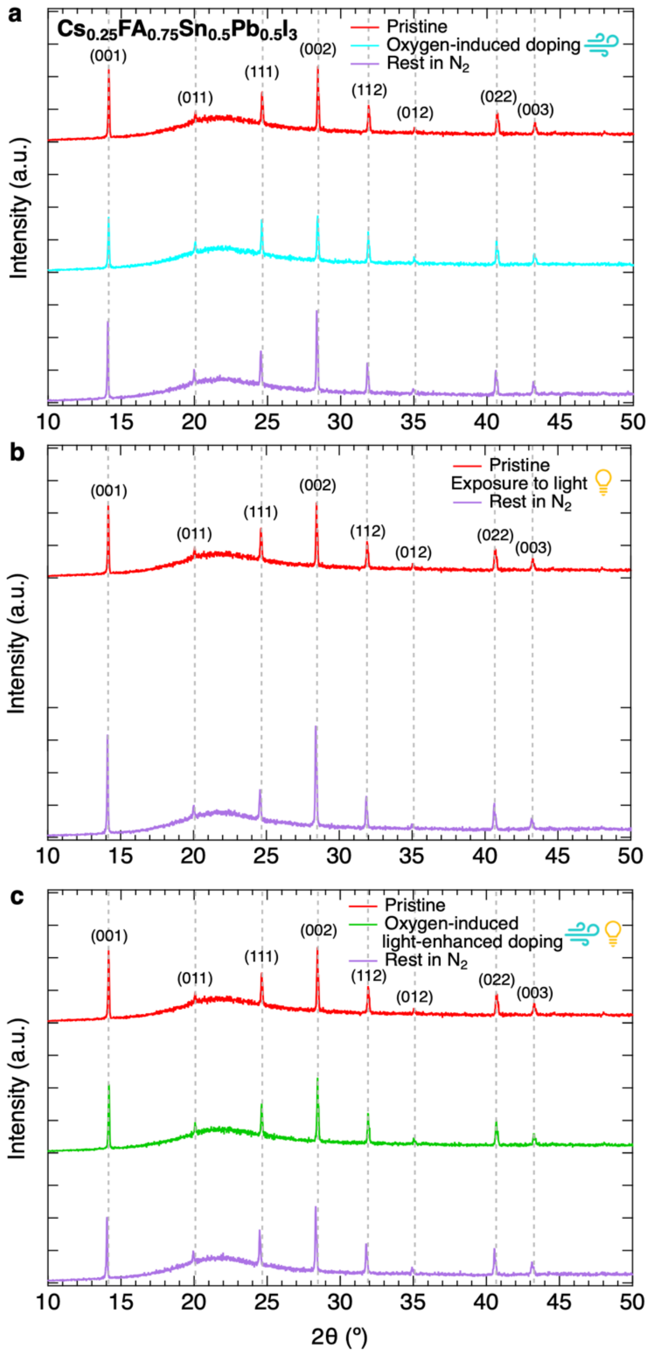

Figure S17: Comparison of XRD patterns of $Sn_{0.5}Pb_{0.5}$ perovskite thin films measured under pristine conditions (in red), after exposure to oxygen for *t* ~ 1 h 30 min (in light blue) or oxygen and light for *t* ~ 15 min (in green) and after resting in N_2 for 1-2 days (in purple). The sample exposed only to light for *t* ~ 30 min was stored in N_2 for ~ 1 h (in purple). The small changes in the XRD peaks intensity are ascribed to slight variations in crystallinity and thickness between the different antisolvent-based spin-coated films. We highlight that these XRD patterns were measured in ambient air. The exposure to ambient air was identical for all samples (hence its effect on the XRD patterns is the same for all) and limited to ~ 10 min.

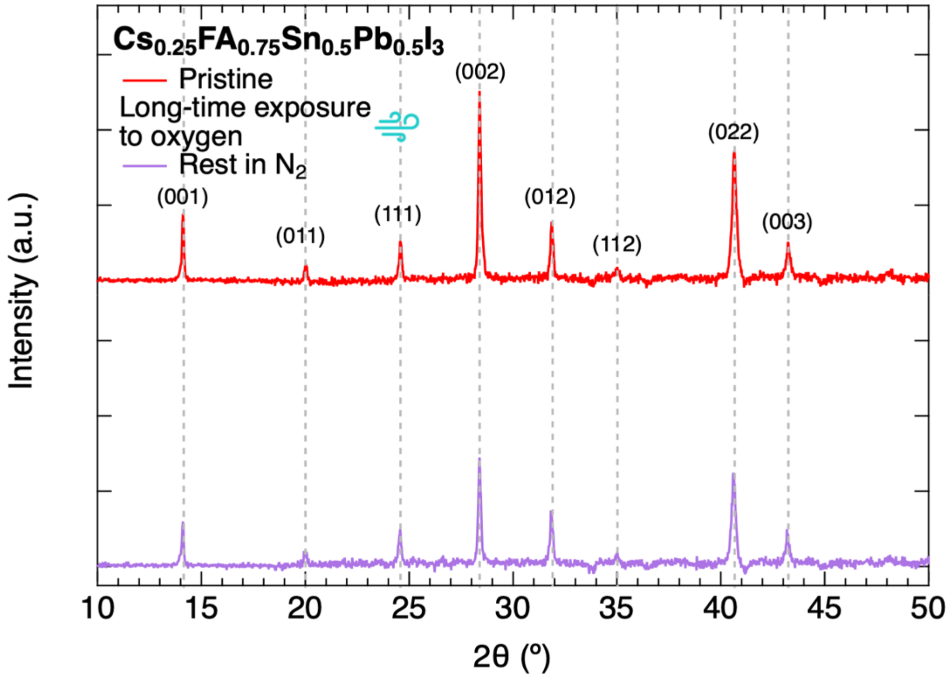

Figure S18: Comparison of structural properties of $Sn_{0.5}Pb_{0.5}$ thin films under pristine conditions (in red) and after exposure to oxygen for *t* ~ 24 h followed by storage in N_2 (in purple), showing the XRD diffraction patterns, corrected with that of the quartz substrate, showing the Miller indices of the characteristic diffraction peaks of the pseudocubic crystal phase. We highlight that these XRD patterns were measured in vacuum.

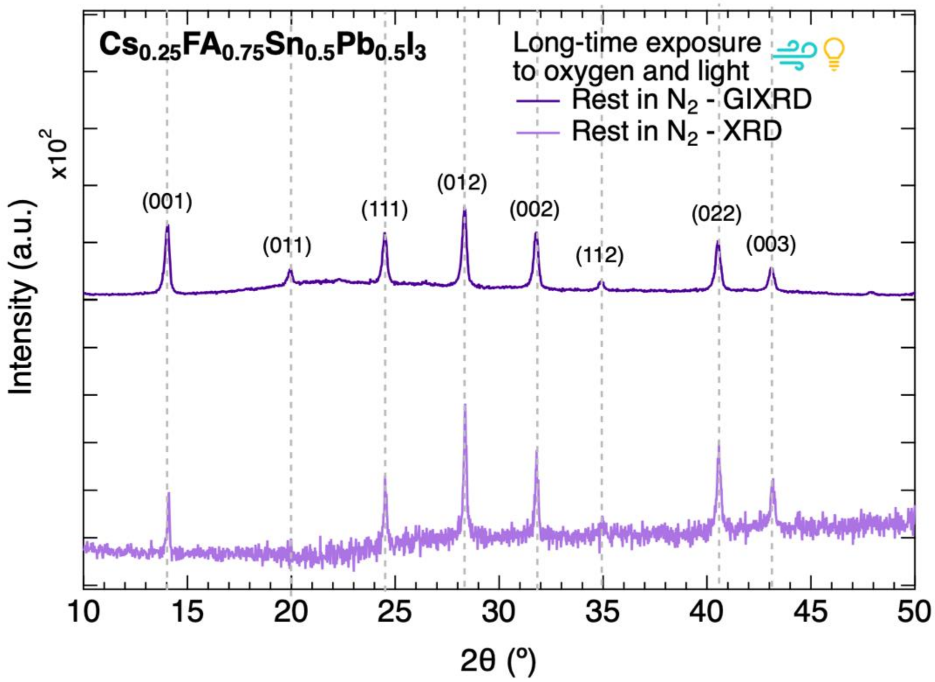

Figure S19: Comparison of XRD and GIXRD diffraction patterns of $Sn_{0.5}Pb_{0.5}$ polycrystalline thin films after prolonged exposure to simultaneously oxygen and light. The GIXRD was performed at an incident angle θ = 0.5º, corresponding to an information depth of ~ 70 nm, namely around one-fourth of the total thickness of the film from the surface. Due to the different measurement parameters, the GIXRD diffraction pattern shows significantly higher intensity, hence its y-axis was rescaled by x10^2. Due to its low intensity, the XRD pattern has been corrected by subtracting the pattern of an empty quartz substrate. We highlight that these XRD patterns were measured in vacuum.

Ultraviolet-visible-near infrared spectroscopy (UV-Vis-NIR) – Optical properties

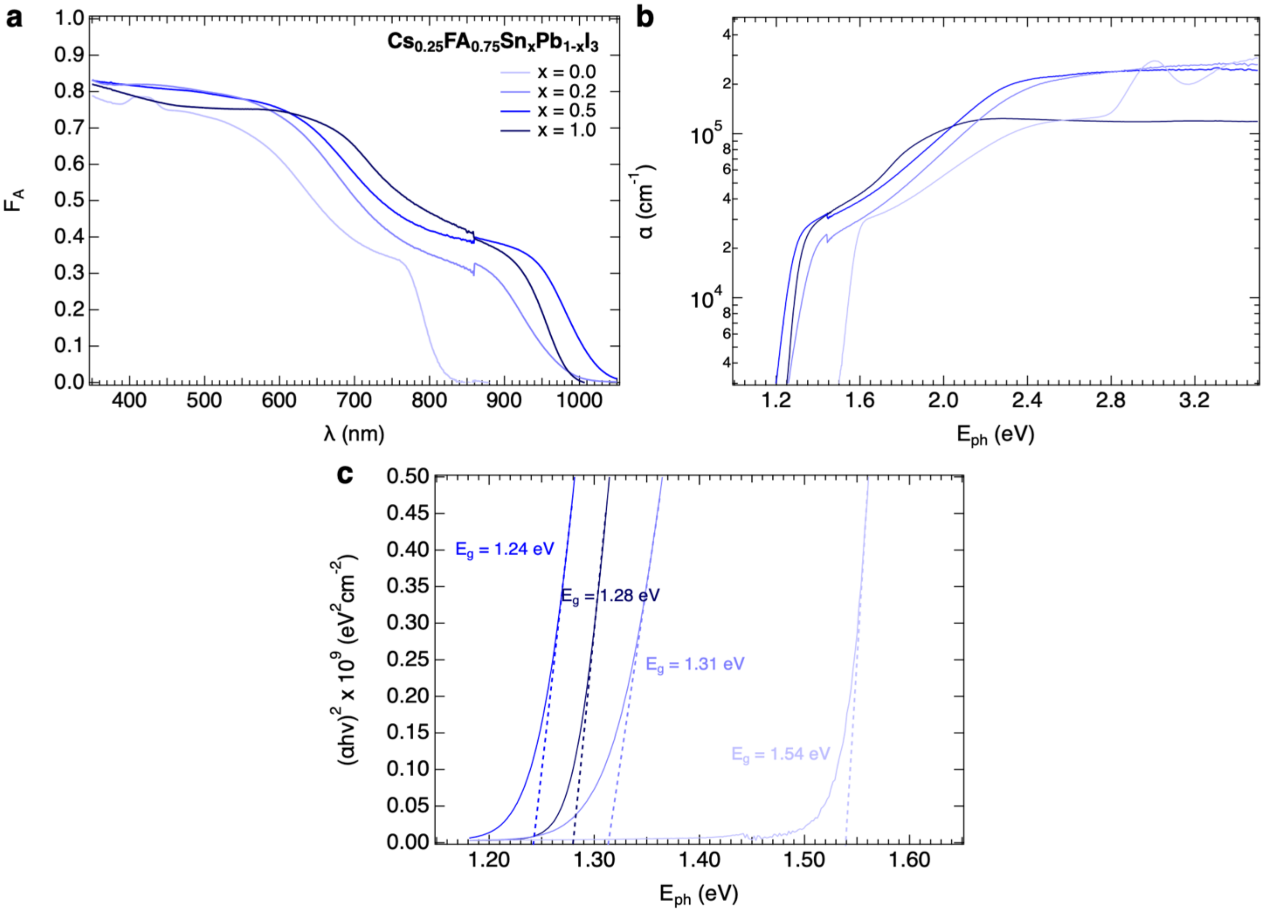

Figure S20: Optical properties of mixed Sn-Pb perovskite thin films of composition $Cs_{0.25}FA_{0.75}Sn_xPb_{1-x}I_3$ with Sn fraction x = 0.0, x = 0.2, x = 0.5 and x = 1.0. (a) Absorptance, i.e. the fraction of absorbed light F_A, spectra and (b) optical bandgaps derived by the linear region of the Tauc plots of the UV-Vis-NIR absorption spectra. Furthermore, the optical bandgaps extracted by the Tauc plots follow the typical bowing behavior depending on the Sn content,[8,9,12] namely the intermediate $Sn_{0.5}Pb_{0.5}$ composition has a lower bandgap E_g ~ 1.24 eV than the extreme compositions Sn_0Pb_1 containing only Pb and Sn_1Pb_0 containing only Sn, which have respectively bandgaps equal to E_g ~ 1.54 eV and E_g ~ 1.28 eV. For $Sn_{0.2}Pb_{0.8}$, the bandgap is E_g ~ 1.31 eV. All these values are in line with those of mixed Sn-Pb perovskites of similar composition.[8,9,11,12,18] The step present in the data at around λ ~ 860 nm (E_{ph} ~ 1.44 eV) is due to the change of detector in the UV-Vis-NIR spectroscopy setup.

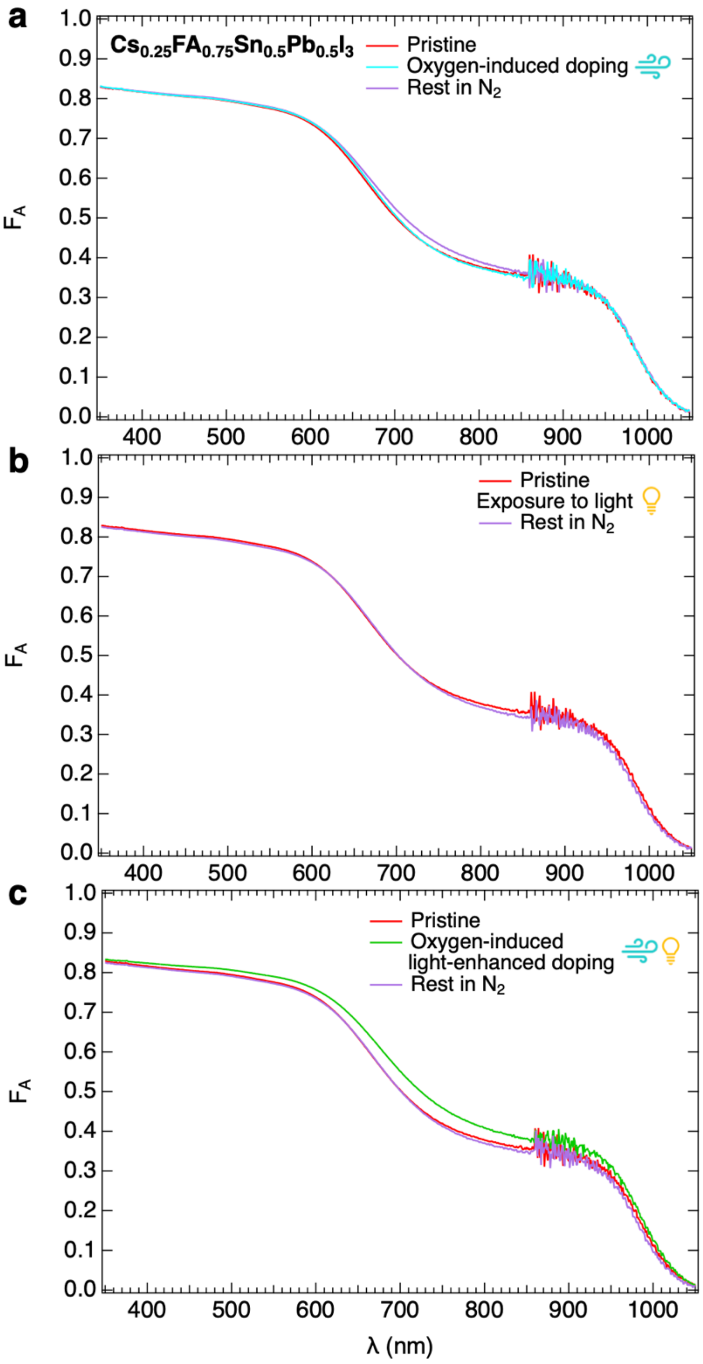

Figure S21: Comparison of absorptance of $Sn_{0.5}Pb_{0.5}$ perovskite thin films measured under pristine conditions (in red), after exposure to oxygen for $t \sim 1$ h 30 min (in light blue) or oxygen and light for $t \sim 15$ min (in green) and after resting in N_2 for 1-2 days (in purple). The sample exposed only to light for $t \sim 30$ min was stored in N_2 for ~ 1 h (in purple). Considering that the oxygen-induced doping corresponds to the change of a minuscule fraction of atoms in the perovskite crystal structure as explained in the main text, it is expected that the absorptance spectra do not change

significantly after exposure to oxygen or oxygen and light or even after resting in N_2. The small changes in the spectra are ascribed to a slight variation in the thickness of the different spin-coated thin films.

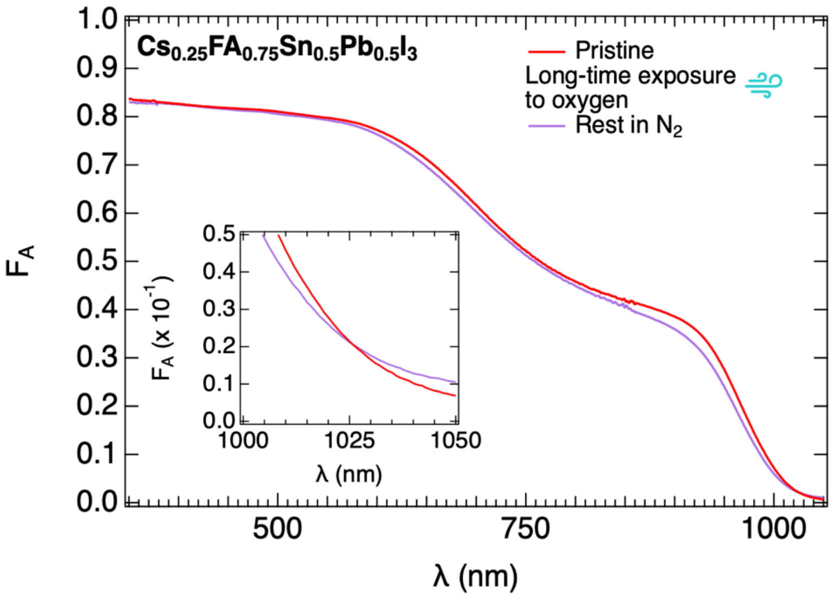

Figure S22: Comparison of optical properties of $Sn_{0.5}Pb_{0.5}$ thin films under pristine conditions (in red) and after exposure to oxygen for *t* ~ 24 h followed by storage in N_2 (in purple). In the inset, focus on the absorptance spectra in the near-infrared spectral region.

X-ray Photoelectron Spectroscopy (XPS) – Elemental composition and depth profiling

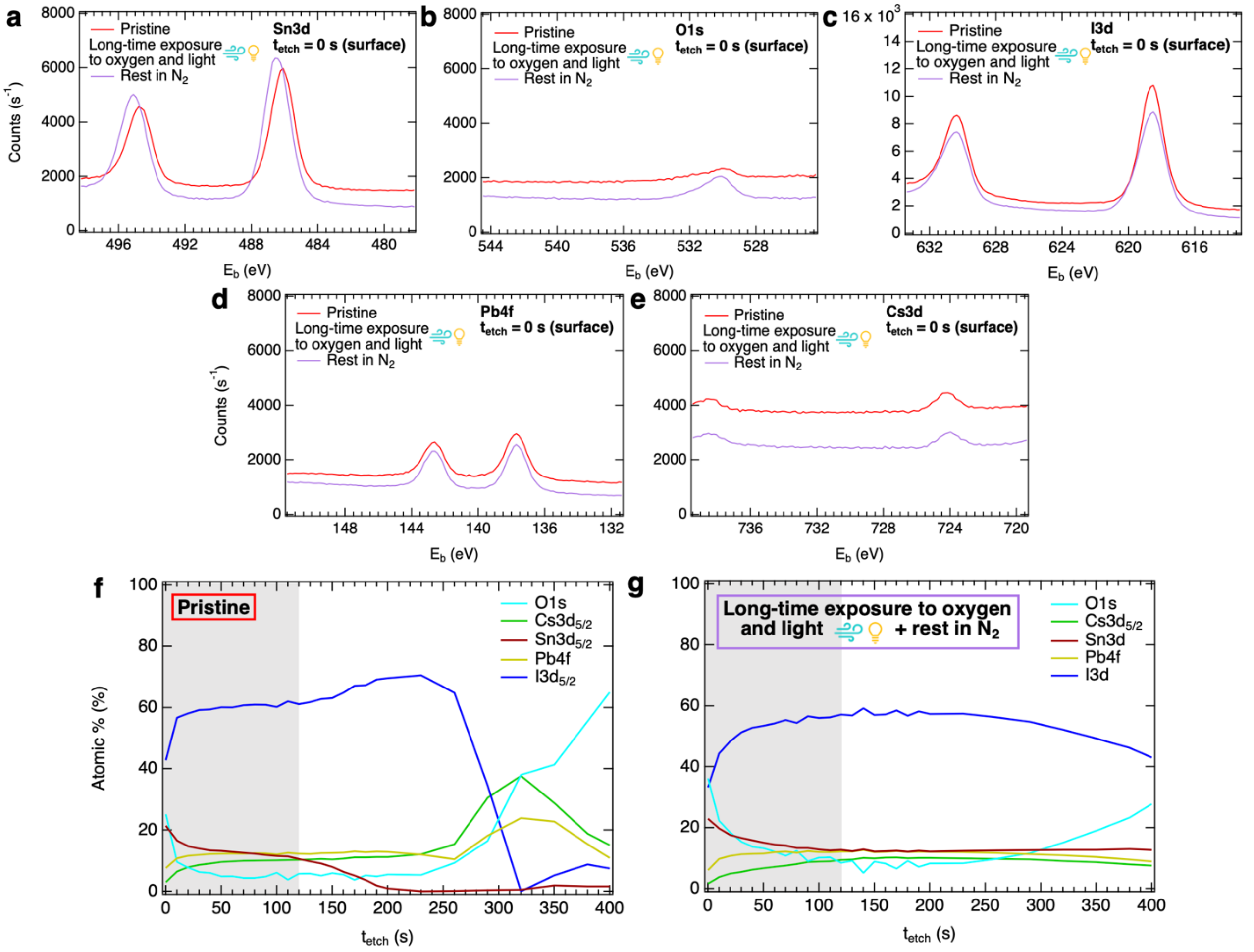

Figure S23: XPS surface analysis presenting the elemental composition, more specifically regarding the (a) Sn3d, (b) O1s, (c) I3d, (d) Pb4f and (e) Cs3d core levels peaks of $Sn_{0.5}Pb_{0.5}$ perovskite thin films under pristine conditions (in red) and after prolonged exposure to simultaneously oxygen and light (in purple). The intensity of the XPS signal for the different electron transitions and elements orbitals is shown as a function of the electron binding energy (E_b). The prolonged exposure to simultaneously oxygen and light leads to a variation in the Sn3d and O1s core levels and a slight change in the I3d ones. Conversely, the Pb4f and Cs3d orbitals seem unchanged. XPS depth profiling of $Sn_{0.5}Pb_{0.5}$ perovskite layers (f) under pristine conditions and (g) after exposure of a film simultaneously to oxygen and light for a long-time interval and subsequent rest in N_2. The relative percentage atomic concentration of O, Cs, Sn, Pb and I elements is shown as a function of etching time (t_{etch}), namely as a function of the probing depth throughout the thickness of the perovskite layer. It must be highlighted that the total percentage of the selected elements adds up to 100% in the measurement, but it does not necessarily correspond to the total makeup of the sample. Interfacial effects probably resulting from the quartz substrate become visible at etching times larger than 300-400 s. The grey shaded area highlights the results of the XPS depth profiling up to 120 s, namely around one fourth of the total thickness of the film, bulk effects closer to the surface of the film can be observed more in detail. A higher Sn/Pb ratio at the film

surface can be noted after exposure of the layer to a combination of oxygen and light. Nevertheless, it is important to emphasize that the compositional dissimilarities in the bulk versus the surface could be due to small differences in crystallization dynamics of Sn- versus Pb-based perovskites during spin-coating.[19-21] However, the Sn enrichment in the first tens of nanometers of the layer demonstrates that the film surface is extremely susceptible to oxidation. Additionally, a subtle depletion of iodide at the film surface can be observed after simultaneous exposure to oxygen and light, which may be due to a partial volatilization of iodide in the form of the oxidation product I_2. These results obtained by XPS are in line with other reports.[22-23]

Table S1: Assignation of XPS peaks at the surface (t_{etch} = 0 s) present at different binding energies to the oxidation species of various elements (Sn, O, I, Pb and Cs) present in the perovskite thin films.

Element core levels	*Oxidation species*	*E_b (eV) - measurements*	*E_b (eV) - literature*
**$Sn3d_{5/2}$**	$Sn^{\delta<2+}$	485.46 (Pristine)	485.20 (Sn^0)[24], 485.60 ($Sn^{\delta<2+}$)[22]
		485.56 (Rest in N_2)	
	Sn^{2+} (PVK) / SnO	486.12 (Pristine)	~ 486.00[24], 486.18,[23] 486.60[22]
		486.30 (Rest in N_2)	
	Sn^{4+} / SnO_2	486.87 (Pristine)	~ 486.60[24], 487.21,[23] 487.40[22]
		487.05 (Rest in N_2)	
**O1s**	Metal oxides / SnO_x	529.86 (Pristine)	529.00-530.00[24], 530.50 (ads species)[22]
		530.09 (Rest in N_2)	
	Organic C=O or metal carbonates	531.19 (Pristine)	531.5-532.00[24], 531.80[22]
		531.44 (Rest in N_2)	
**$I3d_{5/2}$**	I^- (PVK)	618.57 (Pristine)	~ 619 (metal iodides)[24], 619.10 (I^-)[22]
		618.55 (Rest in N_2)	
**$Pb4f_{7/2}$**	Pb^{2+} (PVK)	137.71 (Pristine)	138.00 ($Pb^{\delta<2+}$),[22]138.40[22]
		137.68 (Rest in N_2)	
**$Cs3d_{5/2}$**	Cs^+ (PVK)	724.20 (Pristine)	724 (CsI)[24]
		724.03 (Rest in N_2)	

Table S2: Atomic ratios from XPS depth profiling at different etching times (a) t_{etch} = 0 s, (b) t_{etch} = 10 s and (c) t_{etch} = 120 s. These correspond to different depth levels analyzed through thickness of the perovskite thin film. The Sn:Pb, Sn:I and Sn:O ratios are calculated from the atomic % appearing in the depth profiling considering only the O1s, Cs3d, Sn3d, Pb4f and I3d elements orbitals shown in **Figures S23f** and **S23g**. The Sn^{4+}:Sn^{2+}:$Sn^{\delta<2+}$ and the Other species:SnO_x ratios are calculated by the peak fitting of respectively $Sn3d_{5/2}$ and O1s peaks at t_{etch} = 0 s, namely at the film surface. Such peak fitting for the $Sn3d_{5/2}$ and O1s peaks at t_{etch} = 0 s is shown in the main text.

a

Etching time t_{etch} = 0 s (surface)

Conditions\Ratio	Sn:Pb	Sn:I	Sn:O	Sn^{4+}:Sn^{2+}:$Sn^{\delta<2+}$	Other species:SnO_x
Pristine	1:0.4	1:2	1:1.2	0.1:0.6:0.3	0.6:0.4
Rest in N_2	1:0.3	1:1.5	1:1.6	0.2:0.5:0.3	0.3:0.7

Etching time t_{etch} = 10 s (just below the surface)

Conditions\Ratio	Sn:Pb	Sn:I	Sn:O
Pristine	1:0.7	1:3.4	1:0.6
Rest in N_2	1:0.5	1:2.2	1:1.1

c **Etching time t_{etch} = 120 s (one fourth of the total thickness of the film)**

Conditions\Ratio	Sn:Pb	Sn:I	Sn:O
Pristine	1:1.2	1:5.8	1:0.5
Rest in N_2	1:1	1:4.5	1:0.7

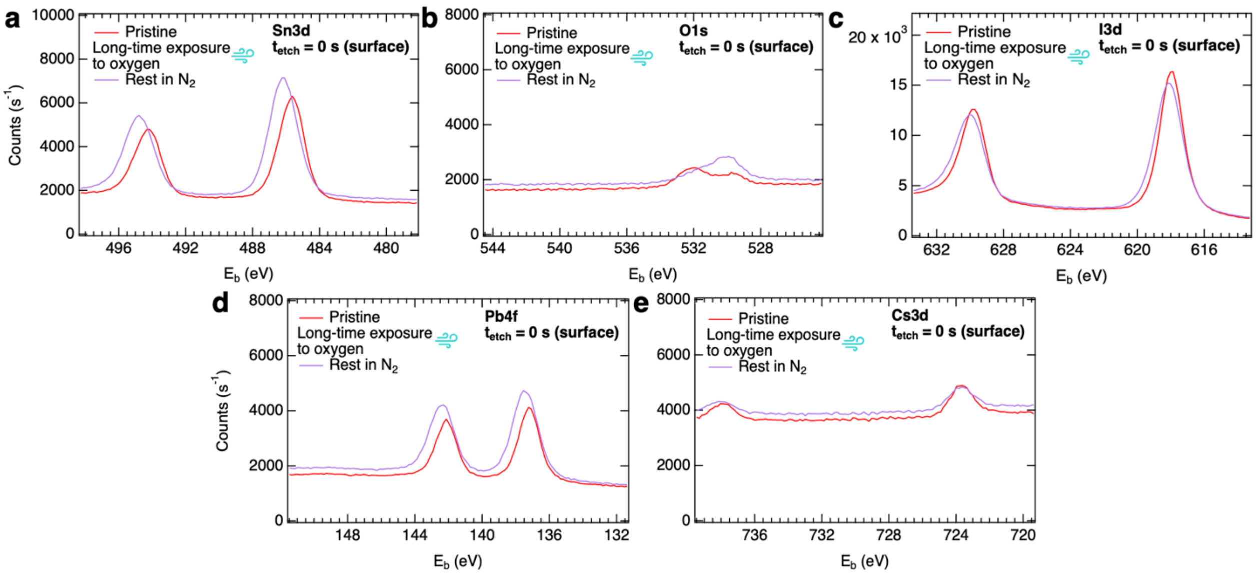

Figure S24: XPS surface analysis presenting the elemental composition, more specifically regarding the (a) Sn3d, (b) O1s, (c) I3d, (d) Pb4f and (e) Cs3d core levels peaks of $Sn_{0.5}Pb_{0.5}$ perovskite thin films under pristine conditions (in red) and after prolonged exposure to oxygen (in purple). The intensity of the XPS signal for the different electron transitions and elements orbitals is shown as a function of the electron binding energy (E_b). Prolonged exposure to oxygen leads to a variation in the Sn3d and O1s core levels, and a slight change in the I3d and Pb4f ones. Conversely, the Cs3d orbitals seem mostly unchanged. These results obtained by XPS are in line with the literature.[22]

Table S3: Assignation of XPS peaks at the surface (t_{etch} = 0 s) present at different binding energies to the oxidation species of various elements (Sn, O, I, Pb and Cs) present in the perovskite thin films.

Element core levels	*Oxidation species*	*E_b (eV) - measurements*	*E_b (eV) - literature*
**$Sn3d_{5/2}$**	Sn	485.64 (Pristine)<br>486.17 (Rest in N_2)	485.20 (Sn^0)[24], 485.60 ($Sn^{\delta<2+}$)[22], ~ 486.00[24], 486.18,[23] 486.60[22] (Sn^{2+}, (PVK) / SnO), ~ 486.60[24], 487.21,[23] 487.40[22] (Sn^{4+} / SnO_2)
**O1s**	O	531.94 (Pristine)<br>530.14 (Rest in N_2)	529.00-530.00 (metal oxides, SnO_x)[24], 530.50 (ads species)[22], 531.5-532.00[24], 531.80[22] (organic C=O or metal carbonates)
**$I3d_{5/2}$**	I^- (PVK)	617.95 (Pristine)<br>618.11 (Rest in N_2)	~ 619 (metal iodides)[24], 619.10 (I^-)[22]
**$Pb4f_{7/2}$**	Pb^{2+} (PVK)	137.19 (Pristine)<br>137.42 (Rest in N_2)	138.00 ($Pb^{\delta<2+}$),[22] 138.40[22], 139.4 ($PbCO_3$)[22]
**$Cs3d_{5/2}$**	Cs^+ (PVK)	723.69 (Pristine)<br>723.70 (Rest in N_2)	724 (CsI)[24]

References

(1) Hutter, E. M. Revealing the Fate of Photo-Generated Charges in Metal Halide Perovskites, Ph.D. Dissertation, Delft University of Technology, Delft, NL, **2018**. DOI: 10.4233/uuid:f8e21539-bd26-4694-b170-6d0641e4c31a

(2) Lal, S. Mixed Tin-Lead Perovskites: Charge Carriers Dynamics and Optical Doping. M.Sc. Thesis, Delft University of Technology, Delft, NL, **2022**.

(3) *Reference Air Mass 1.5 Spectra*. (n.d.). Grid Modernization | NREL. https://www.nrel.gov/grid/solar-resource/spectra-am1.5.html (accessed October 16, **2022**).

(4) Zhao, J., Van Der Poll, L. M., Looman, S. L., Yan, J., Thieme, J., Ibrahim, B., & Savenije, T. J. Long-Lived Charge Extraction in CsMAFA-Based Perovskites in n-i-p and p-i-n Structures. *ACS Energy Letters* **2024**, 9(5), 2456–2463. DOI: 10.1021/acsenergylett.4c00250

(5) Neamen, D. A. (**2012**). *Semiconductor Physics and Devices: Basic Principles* (pp. 107-118). *McGraw-Hill Education*.

(6) Konstantakou, M., & Stergiopoulos, T. A critical review on tin halide perovskite solar cells. *Journal of Materials Chemistry A* **2017,** *5*(23), 11518–11549. DOI: 10.1039/c7ta00929a

(7) Savill, K. J., Ulatowski, A. M., & Herz, L. M. Optoelectronic Properties of Tin–Lead Halide Perovskites. *ACS Energy Letters* **2021**, *6*(7), 2413–2426. DOI: 10.1021/acsenergylett.1c00776

(8) Savill, K. J., Ulatowski, A. M., Farrar, M. D., Johnston, M. B., Snaith, H. J., & Herz, L. M. Impact of Tin Fluoride Additive on the Properties of Mixed Tin-Lead Iodide Perovskite Semiconductors. *Advanced Functional Materials* **2020**, *30*(52), 2005594. DOI: 10.1002/adfm.202005594

(9) Zong, Y., Wang, N., Zhang, L., Ju, M., Zeng, X. C., Sun, X. W., Zhou, Y., & Padture, N. P. Homogenous Alloys of Formamidinium Lead Triiodide and Cesium Tin Triiodide for Efficient Ideal-Bandgap Perovskite Solar Cells. *Angewandte Chemie International Edition* **2017**, *56*(41), 12658–12662. DOI: 10.1002/anie.201705965

(10) Cullity, B. D. *Elements of X-Ray Diffraction (Addison-Wesley series in metallurgy and materials)*(2nd ed.). Addison-Wesley, Boston, **1978**.

(11) Klug, M. T., Milot, R. L., Patel, J. B., Green, T., Sansom, H. C., Farrar, M. D., Ramadan, A. J., Martani, S., Wang, Z., Wenger, B., et al. Metal composition influences optoelectronic quality in mixed-metal lead–tin triiodide perovskite solar absorbers. *Energy & Environmental Science* **2020**, *13*(6), 1776–1787. DOI: 10.1039/d0ee00132e

(12) Eperon, G. E., Leijtens, T., Bush, K. A., Prasanna, R., Green, T., Wang, J. T. W., McMeekin, D. P., Volonakis, G., Milot, R. L., May, R., et al. Perovskite-perovskite tandem photovoltaics with optimized band gaps. *Science* **2016**, *354*(6314), 861–865. DOI: 10.1126/science.aaf9717

(13) Hoefler, S. F., Trimmel, G., & Rath, T. Progress on lead-free metal halide perovskites for photovoltaic applications: a review. *Monatshefte Für Chemie - Chemical Monthly* **2017**, *148*(5), 795–826. DOI: 10.1007/s00706-017-1933-9

(14) Holder, C. F., & Schaak, R. E. Tutorial on Powder X-ray Diffraction for Characterizing Nanoscale Materials. *ACS Nano* **2019**, *13*(7), 7359–7365. DOI: 10.1021/acsnano.9b05157

(15) Wang, J., Gao, Z., Yang, J., Lv, M., Chen, H., Xue, D., Meng, X., & Yang, S. Controlling the Crystallization Kinetics of Lead-Free Tin Halide Perovskites for High Performance Green Photovoltaics. *Advanced Energy Materials* **2021**, *11*(39), 2102131. DOI: 10.1002/aenm.202102131

(16) Pitaro, M., Tekelenburg, E. K., Shao, S., & Loi, M. A. Tin Halide Perovskites: From Fundamental Properties to Solar Cells. *Advanced Materials* **2021**, *34*(1), 2105844. DOI: 10.1002/adma.202105844

(17) Cao, J., Loi, H., Xu, Y., Guo, X., Wang, N., Liu, C., Wang, T., Cheng, H., Zhu, Y., Li, M. G., et al. High-Performance Tin–Lead Mixed-Perovskite Solar Cells with Vertical Compositional Gradient. *Advanced Materials* **2021**, *34*(6), 2107729. DOI: 10.1002/adma.202107729

(18) Prasanna, R., Gold-Parker, A., Leijtens, T., Conings, B., Babayigit, A., Boyen, H. G., Toney, M. F., & McGehee, M. D. Band Gap Tuning via Lattice Contraction and Octahedral Tilting in Perovskite Materials for Photovoltaics. *Journal of the American Chemical Society* **2017**, *139*(32), 11117–11124. DOI: 10.1021/jacs.7b04981

(19) Cao, J., Loi, H., Xu, Y., Guo, X., Wang, N., Liu, C., Wang, T., Cheng, H., Zhu, Y., Li, M. G., et al. High-Performance Tin–Lead Mixed-Perovskite Solar Cells with Vertical Compositional Gradient. *Advanced Materials* **2021**, *34*(6), 2107729. DOI: 10.1002/adma.202107729

(20) Wang, J., Gao, Z., Yang, J., Lv, M., Chen, H., Xue, D., Meng, X., & Yang, S. Controlling the Crystallization Kinetics of Lead-Free Tin Halide Perovskites for High Performance Green Photovoltaics. *Advanced Energy Materials* **2021**, *11*(39), 2102131. DOI: 10.1002/aenm.202102131

(21) Pitaro, M., Tekelenburg, E. K., Shao, S., & Loi, M. A. Tin Halide Perovskites: From Fundamental Properties to Solar Cells. *Advanced Materials* **2021**, *34*(1), 2105844. DOI: 10.1002/adma.202105844

(22) Mundt, L. E., Tong, J., Palmstrom, A. F., Dunfield, S. P., Zhu, K., Berry, J. J., Schelhas, L. T., & Ratcliff, E. L. Surface-Activated Corrosion in Tin–Lead Halide Perovskite Solar Cells. *ACS Energy Letters* **2020**, *5*(11), 3344–3351. DOI: 10.1021/acsenergylett.0c01445

(23) Lanzetta, L., Webb, T., Zibouche, N., Liang, X., Ding, D., Min, G., Westbrook, R. J. E., Gaggio, B., Macdonald, T. J., Islam, M. S., et al. Degradation mechanism of hybrid tin-based perovskite solar cells and the critical role of tin (IV) iodide. *Nature Communications* **2021**, *12*(1), 2853. DOI: 10.1038/s41467-021-22864-z

(24) *Table of Elements | Thermo Fisher Scientific - IE*. (n.d.). https://www.thermofisher.com/nl/en/home/materials-science/learning-center/periodic-table.html (accessed February 8, **2023**).
